# Epigenetic Control of Effector Gene Expression in the Plant Pathogenic Fungus *Leptosphaeria maculans*

**DOI:** 10.1371/journal.pgen.1004227

**Published:** 2014-03-06

**Authors:** Jessica L. Soyer, Mennat El Ghalid, Nicolas Glaser, Bénédicte Ollivier, Juliette Linglin, Jonathan Grandaubert, Marie-Hélène Balesdent, Lanelle R. Connolly, Michael Freitag, Thierry Rouxel, Isabelle Fudal

**Affiliations:** 1 INRA, UR 1290 BIOGER-CPP, Thiverval-Grignon, France; 2 Department of Biochemistry and Biophysics, Center for Genome Research and Biocomputing, Oregon State University, Corvallis, Oregon, United States of America; University of Exeter, United Kingdom

## Abstract

Plant pathogens secrete an arsenal of small secreted proteins (SSPs) acting as effectors that modulate host immunity to facilitate infection. SSP-encoding genes are often located in particular genomic environments and show waves of concerted expression at diverse stages of plant infection. To date, little is known about the regulation of their expression. The genome of the Ascomycete *Leptosphaeria maculans* comprises alternating gene-rich GC-isochores and gene-poor AT-isochores. The AT-isochores harbor mosaics of transposable elements, encompassing one-third of the genome, and are enriched in putative effector genes that present similar expression patterns, namely no expression or low-level expression during axenic cultures compared to strong induction of expression during primary infection of oilseed rape (*Brassica napus*). Here, we investigated the involvement of one specific histone modification, histone H3 lysine 9 methylation (H3K9me3), in epigenetic regulation of concerted effector gene expression in *L. maculans*. For this purpose, we silenced the expression of two key players in heterochromatin assembly and maintenance, *HP1* and *DIM-5* by RNAi. By using HP1-GFP as a heterochromatin marker, we observed that almost no chromatin condensation is visible in strains in which *LmDIM5* was silenced by RNAi. By whole genome oligoarrays we observed overexpression of 369 or 390 genes, respectively, in the silenced-*LmHP1* and -*LmDIM5* transformants during growth in axenic culture, clearly favouring expression of SSP-encoding genes within AT-isochores. The ectopic integration of four effector genes in GC-isochores led to their overexpression during growth in axenic culture. These data strongly suggest that epigenetic control, mediated by HP1 and DIM-5, represses the expression of at least part of the effector genes located in AT-isochores during growth in axenic culture. Our hypothesis is that changes of lifestyle and a switch toward pathogenesis lift chromatin-mediated repression, allowing a rapid response to new environmental conditions.

## Author Summary

Effectors are key players in pathogenicity of microbes toward plants. Effector genes usually show concerted expression during plant infection but how this concerted expression is generated remains a largely unexplored research topic. Epigenetic mechanisms are involved in genome maintenance and integrity but are increasingly considered as important for regulation of gene expression in numerous and diverse organisms. Here we show that the genomic environment has impact on expression of *Leptosphaeria maculans* effector genes, and that an epigenetic mechanism that relies on two proteins involved in heterochromatin formation and maintenance, HP1 and DIM-5, modulates this expression, leading to repression during growth in axenic culture. Chromatin decondensation by removal of histone H3 lysine 9 methylation and/or HP1 is presumably a prerequisite for effector gene expression during primary infection of oilseed rape. Thus we show chromatin-based transcriptional regulation that can act on effector gene expression in fungi. Our study highlights the importance of heterochromatic landscapes, not only for genome maintenance but also in rapid and efficient adaptation of organisms to changing environmental situations.

## Introduction

During infection, plant pathogenic microbes secrete a set of molecules collectively known as effectors, pathogenicity determinants that modulate plant innate immunity and enable parasitic infection [1]. Effectors are either targeted to the apoplast or the cytoplasm of the plant where they are mainly involved in overcoming the host immune defence system, nutrient uptake and, eventually, symptom development [2]. In many plant pathogenic microbes, effectors show common features. They are small proteins, potentially secreted (as many possess signal peptide sequences), generally cysteine-rich and usually have no homology to known proteins in databases [3]. Thus, these proteins are referred to as Small Secreted Proteins (SSPs). Bioinformatic analyses based on common features of effectors aim to identify the complete repertoire of candidate effector genes of several oomycetes [4], [5] and filamentous fungi [6]–[8]. These studies highlighted another common trait of effector genes: their concerted up-regulation upon plant infection which suggests co-operation between a subset of effectors at different time points [9], [10].

While high throughput functional analyses of effectors are in progress in several research groups (*e.g.* in *Ustilago maydis* [6], in *Magnaporthe oryzae* [11] and in *Phytophthora infestans* [12]), less effort has focused on the regulation of effector gene expression. Effector genes of filamentous plant pathogens are often located close to dispersed transposable elements (TEs) or in TE-rich regions of the genome, like dispensable chromosomes or telomeres [13]. For example, in *Leptosphaeria maculans*, the effector gene encoding AvrLm11 was found to be located on a conditionally dispensable chromosome (CDC) [14], while the avirulence gene of *M. oryzae Avr-Pita* is located in a sub-telomeric region [15]. In *Fusarium oxysporum*, all known effector genes are located in one of its four dispensable chromosomes [16], and in *P. infestans* effector genes are located in highly plastic genomic regions, enriched in TEs [5]. The location of effector genes in dynamic genomic regions, enriched in TEs is suggested to have an impact on adaptability to new host plants [17], [18]. Here we postulate that it could also influence their expression.

*L. maculans* is an ascomycete fungus belonging to the Dothideomycete class that causes stem canker of oilseed rape (*Brassica napus*). Sequencing of the *L. maculans* genome has revealed an unusual genome structure compared to other fungi as it contains alternating GC-equilibrated (∼51% GC) and AT-rich (∼33.9% GC) blocks, which we call “GC-” and “AT-isochores”. The GC-isochores are enriched in housekeeping genes whereas the AT-isochores are gene-poor, enriched in TEs truncated and degenerated by Repeat-Induced Point mutation (RIP) and comprise 36% of the genome but only 5% of the genes [7]. One hundred and twenty-two (∼20%) of all genes encoding SSPs have been identified in the AT-isochores [7] including the CDC of *L. maculans*. These genes include five with experimentally demonstrated effector activity (*AvrLm1*, *AvrLm6*, *AvrLm4-7*, *LmCys2* and *AvrLm11*; [14], [19]–[21], and our unpublished data). These effector genes share similar expression profiles, namely very low expression in axenic culture and a drastic increase in expression during primary leaf infection, with a peak of expression 7 days post inoculation (dpi). This expression pattern is a common characteristic of SSP-encoding genes located in AT-isochores, as 73% of these genes are overexpressed at 7 dpi (compared to 19% of SSP-encoding genes in GC-isochores) when compared to growth in axenic culture [7]. AT-isochores in *L. maculans* were postulated to be heterochromatic regions because they share characteristics of heterochromatin in many organisms: (i) they are rich in TEs affected by RIP [7], [22], [23]), (ii) they are gene poor and (iii) they show lower rates of recombination compared to GC-isochores [7]. Based on this, we speculated that AT-isochores may be targets of reversible epigenetic modifications that affect the regulation of effector genes and would thus be instrumental in concerted expression at the onset of plant infection.

Most eukaryotic DNA is associated with histones and non-histone proteins to form chromatin. At least two different states of chromatin are commonly distinguished: gene-rich euchromatin and gene-poor heterochromatin [24]. Euchromatin is less condensed, more accessible and generally more easily transcribed than heterochromatin. The latter is highly condensed and less accessible to the transcriptional machinery. Additionally, heterochromatin is discriminated into “constitutive heterochromatin”, mainly found at centromeres, sub–telomeres and rDNA clusters, and is thought to be involved in genome stability and integrity, and “facultative heterochromatin” that is dispersed throughout the genome. Facultative heterochromatin has attracted considerable attention, as it allows for epigenetic regulation of gene expression by changing chromatin states. Chromatin states can be classified by distinct combinations of post-translational modifications targeted to histones, commonly referred to as the “histone code” [25], [26]. Heterochromatin is enriched in specific modifications, which are thought to be heritable and reversible, and thus called “epigenetic”. These modifications include lysine hypoacetylation of histone H3 and H4, as well as trimethylation of H3 lysine 9 (H3K9me3) and lysine 27 (H3K27me3) [27]. Other hallmarks are presence of Heterochromatin Protein 1 (HP1) and cytosine DNA methylation [24]. In fungi, mechanisms of heterochromatin formation have been investigated mostly in *Neurospora crassa*, where H3K9me3 is catalysed by DIM-5 [28], [29]. The HP1 ortholog of *N. crassa* binds to H3K9me3 *via* its chromo domain and recruits the DNA methyltransferase DIM-2 *via* HP1 chromo-shadow domain, resulting in DNA methylation in essentially all AT-rich heterochromatic regions [28]–[32].

On the basis of the specific location of effector genes in AT-isochores of the genome of *L. maculans*, we investigated whether the concerted expression of these effector genes is influenced by their genomic environment. We generated *L. maculans* transformants in which expression of the *L. maculans* orthologs of *DIM-5 and HP1* (*LmDIM5* and *LmHP1*) was decreased, analysed chromatin states in the transformants by cytology and performed oligoarray experiments to study the influence of LmHP1 and LmDIM5 on gene expression, with a focus on effector genes. We also analysed effects of changing genomic environment from AT- to GC-isochores on effector gene expression. Our data showed HP1- and DIM-5-mediated repression of effector genes during growth in axenic culture and strongly suggest an epigenetic mechanism is the basis of concerted expression of effector genes during plant infection.

## Results

### Identification and RNAi silencing of orthologs of *HP1* and *DIM-5* in *L. maculans*

**Figure 1:**
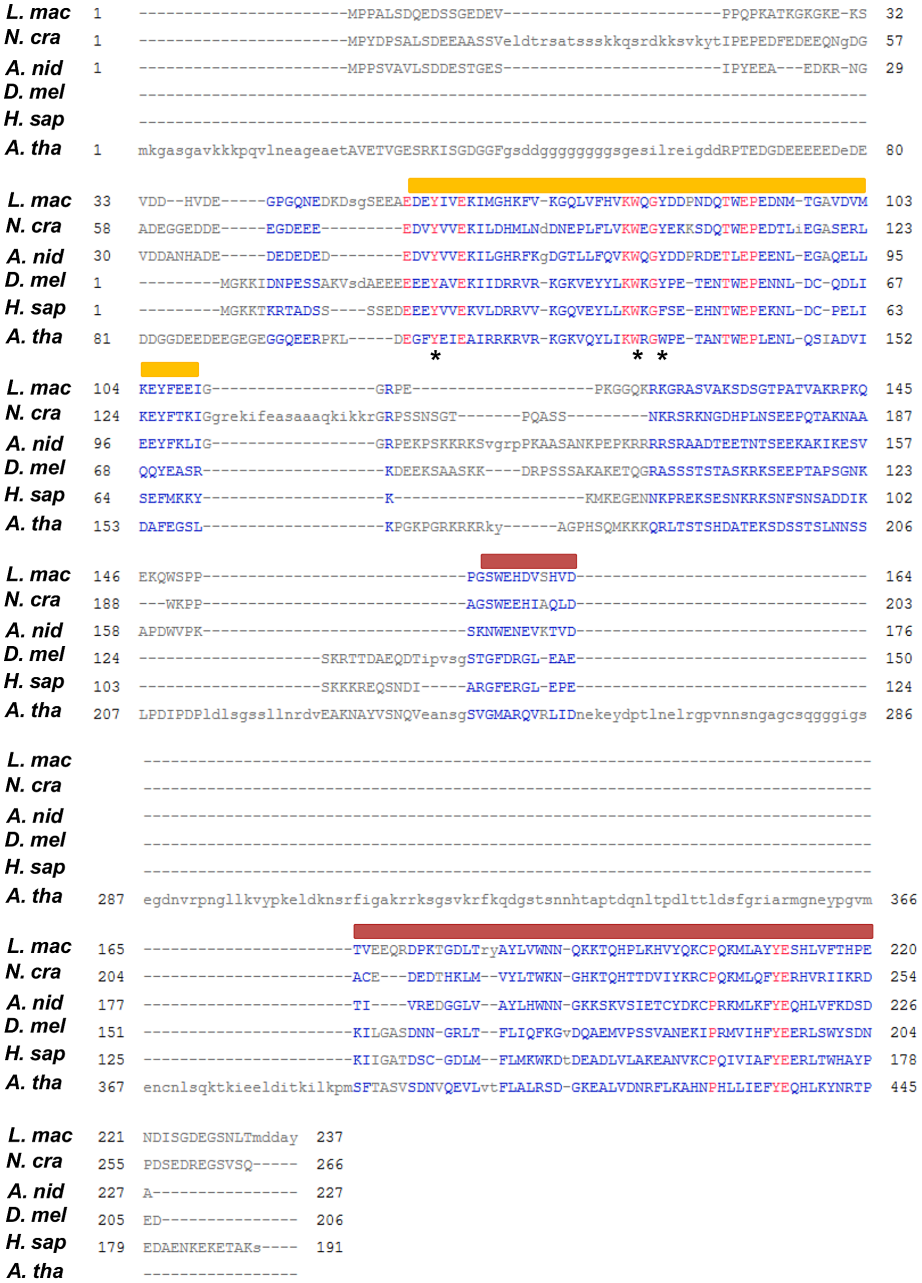
Multiple sequence alignment of LmHP1 with other characterised HP1 proteins. Amino acid sequences were retrieved from the Entrez database of NCBI. *L. mac*, *L. maculans* CBX96122.1; *A. nid*, *Aspergillus nidulans* CBF85797.1; *N. cra*, *Neurospora crassa* AAR19291.1; *D. mel*, *Drosophila melanogaster* AAA28620.1; *H. sap*, *Homo sapiens* NP_001120794.1; *A. tha*, *A. thaliana* AED92456.1. The orange bar indicates the conserved chromo domain involved in the recognition of H3K9me3 and also H3K27me3 in *A. thaliana*; the red bar indicates the conserved chromo-shadow domain involved in protein-protein interactions [33]. The asterisks indicate the three aromatic residues forming the binding pocket for H3K9me3 [54].

**Figure 2:**
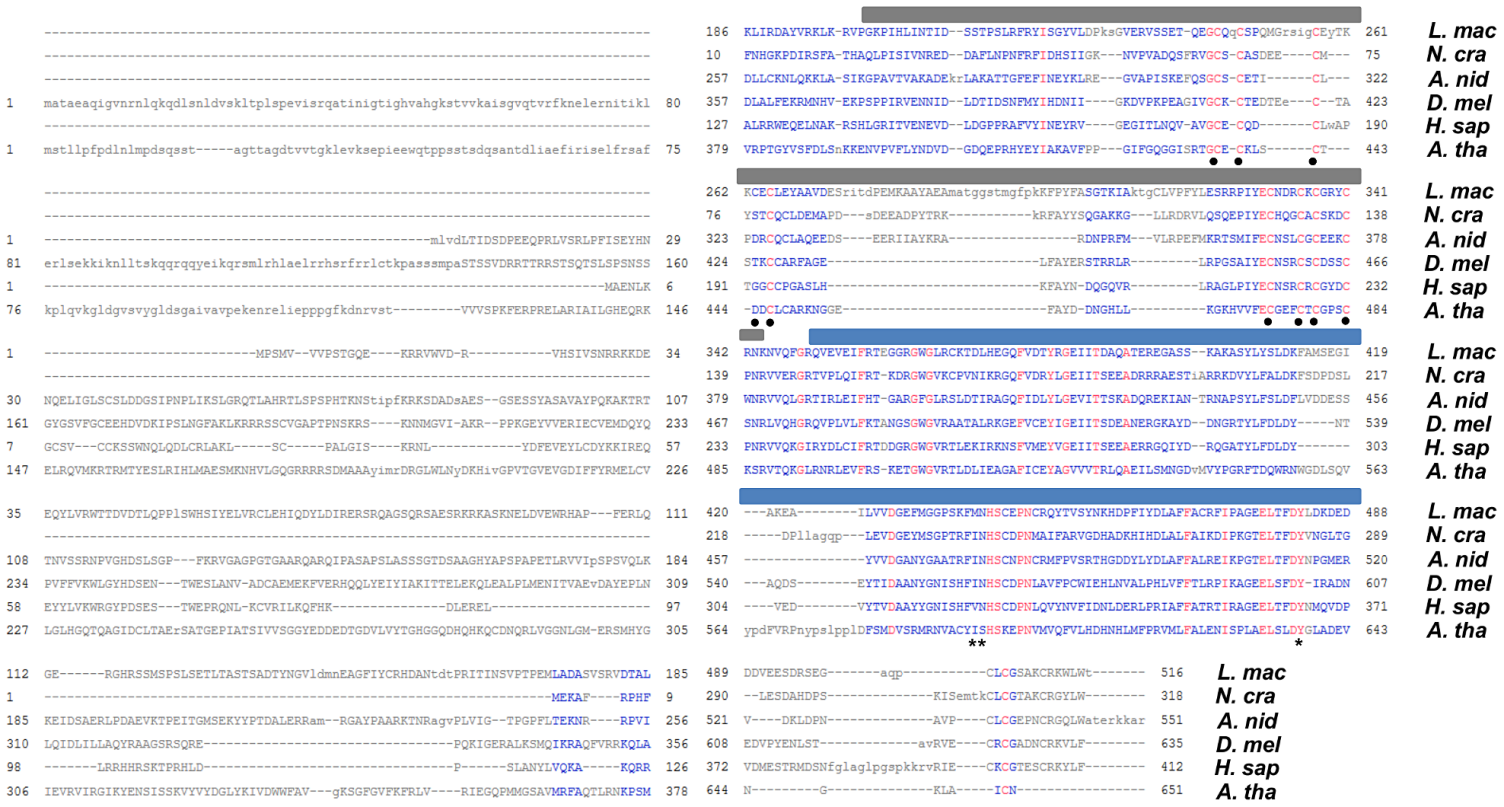
Multiple sequence alignment of LmDIM5 with other characterised DIM-5 proteins. Amino acid sequences were retrieved from the Entrez database of NCBI. Alignment of DIM-5 proteins; *L. mac*, *L. maculans* CBX92341.1; *A. nid*, *Aspergillus nidulans* CBF88005.1; *N. cra*, *Neurospora crassa* AAL35215.1; *D. mel*, *Drosophila melanogaster* AAF55154.1; *H. sap*, *Homo sapiens* CAG46546.1; *A. tha*, *A. thaliana* AAK28967.1. The grey bar indicates the conserved pre-SET domain that includes a Zn_3_Cys_9_ motif; the blue bar indicates the conserved SET-domain involved in AdoMet binding site and active site. The nine cysteines residues forming the conserved zinc cluster are indicated with black dots; the three residues with catalytic roles are indicated with asterisks [35].

Orthologs of *N. crassa* DIM-5 and HP1 were identified in *L. maculans* by bidirectional best hits with BLASTp [28], [30]. *LmHP1* encodes a protein of 237 amino acids (37% of all residues are identical to *N. crassa* HP1), containing the typical N-terminal chromo domain (IPR000953) and the C-terminal chromo-shadow domain (IPR008251; Figure 1) [33]. *LmHP1* was further annotated by RACE-PCR. Five introns, a 5′UTR of 140 bp and a 3′UTR of 444 bp were identified (GenBank accession number CBX96122). *LmDIM5* encodes a protein of 516 amino acids (36% identity with *N. crassa* DIM-5). This family of Su(var)3–9 histone methyltransferases was first identified in *Drosophila melanogaster* [34] and is evolutionarily conserved. LmDIM5 exhibits the two typical domains of DIM-5 proteins described so far, a central Pre-SET (or CXC) domain (IPR007728) and a C-terminal SET domain (IPR001214) (Figure 2) [35]. The N-terminal region of DIM-5 is less conserved (Figure 2), as some proteins (*i.e.* Su[var]3–9 from *D. Melanogaster*, *Homo sapiens* and Clr4 from *Schizosaccharomyces pombe* [34], [36], [37]) have a chromo domain, which is absent from the protein in filamentous ascomycetes (Figure 2). Six introns, a 5′UTR of 259 bp and a 3′UTR of 262 bp were identified in *LmDIM5* by RACE-PCR (GenBank accession number CBX92341).

**Figure 3:**
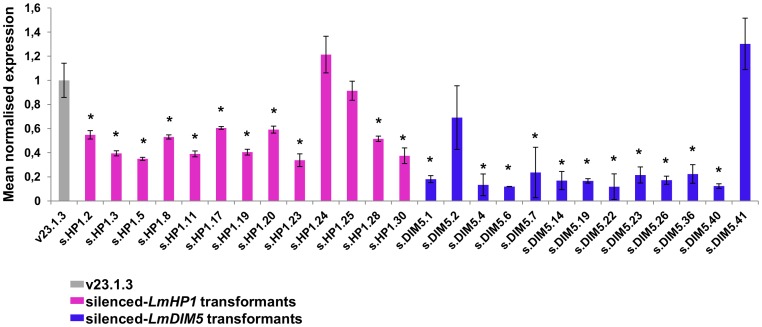
Levels of expression of *LmHP1* and *LmDIM5* in *L. maculans* isolate v23.1.3 silenced by RNAi. The vector pPZPnat1-*LmHP1* and pPZPnat1-*LmDIM5* were transformed, independently, into the wild type strain v23.1.3 *via A. tumefaciens*-mediated transformation (ATMT). Thirty and 45 transformants resistant to nourseothricin were recovered respectively. Among them, 13 transformants were tested for the residual expression of either *LmHP1* or *LmDIM5* gene by qRT-PCR. Expression is relative to the 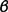 *-Lmtubulin* expression level and to the expression of *LmHP1* and *LmDIM5* in the wild-type isolate v23.1.3 (grey bar; 2^−ΔΔCt^ method); expression of *Lmactin* relative to 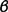 *-Lmtubulin* was used as control. Four silenced-*LmHP1* and four silenced-*LmDIM5* transformants were selected for further characterisation (s.HP1.3, .5, 17 and .19; s.DIM5.1, .4, .19 and .40). For each gene, a transformant that has not been silenced was also selected (s.HP1.25 and s.DIM5.41) as a control. RNA has been extracted from mycelial culture; each data point is the average of two biological repeats (two extractions from different biological material) and two technical repeats (two RT-PCRs). Error bars indicate the standard deviation of two biological and technical repeats. Probability: * indicates *P*<0.05.

*LmHP1* and *LmDIM5* were silenced by RNAi. While all primary *LmDIM5* transformants grew, only one-third (30/89) of the *LmHP1* transformants were able to grow after primary transformants were plated on a second round of selection medium. Expression of *LmHP1* and *LmDIM5* during mycelial growth was determined by qRT-PCR in a subset of transformants (Figure 3). Six transformants showed only 12–20% of *LmDIM5* expression compared to the wild type strain, while expression of *LmHP1* was never lower than 33% of wild type expression (Figure 3). Five transformants per construct were selected for further characterisation, four transformants with significant levels of silencing and one control transformant each for which *LmHP1* or *LmDIM5* expression was similar to that of wild type.

### Silencing of *LmDIM5* and *LmHP1* result in growth defects but only *LmDIM5* silencing reduces pathogenicity

**Figure 4:**
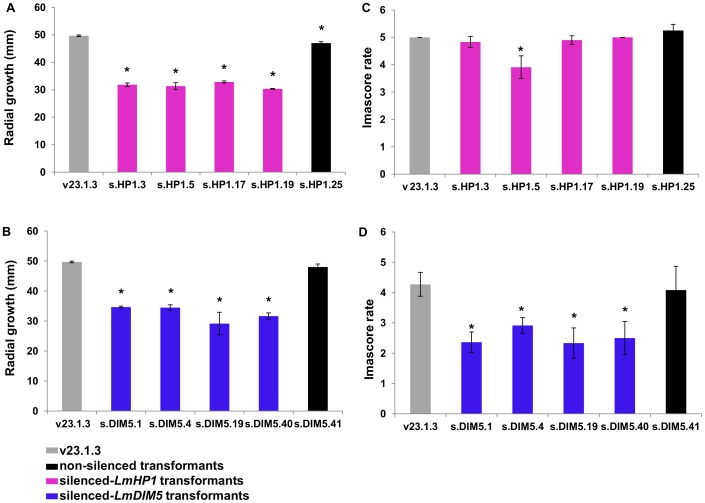
Effect of *LmHP1* and *LmDIM5* silencing on growth and pathogenicity of *L. maculans*. Radial growth and pathogenicity were assessed in the transformants and the wild type strain v23.1.3. Grey: wild type strain; black: non-silenced transformant; purple bars: silenced-*LmHP1* transformants and blue bars: silenced-*LmDIM5* transformants. (A) and (B) radial growth (mm) of the selected silenced*-LmHP1* or silenced*-LmDIM5* transformants (Figure 3) 10 days post inoculation on V8 agar plates. (C) and (D) pathogenicity assays of the selected silenced*-LmHP1* and silenced*-LmDIM5* transformants 14 days after inoculation on the susceptible cultivar Westar of *B. napus*. Results are expressed as the mean scoring using the IMASCORE rating scale comprising six infection classes (IC), where IC1 to IC3 corresponded to resistance and IC4 to IC6, to susceptibility [89]. Error bars indicate the standard deviation of two biological and technical repeats. Probability: * indicates *P*<0.05.

Transformants in which either *LmHP1* or *LmDIM5* were silenced showed an average linear growth reduction of 34% in axenic culture when compared to wild type and non-silenced transformants (Figures 4A and 4B). These transformants were not affected in sexual reproduction when crossed with strain v24.1.2 (data not shown). No pathogenicity defect was associated with silencing of *LmHP1* (Figure 4C) but silencing of *LmDIM5* resulted in reduced pathogenicity (Figure 4D).

### Chromatin decondensation is observed in nuclei of silenced-*LmDIM5* transformants during mycelial growth

**Figure 5:**
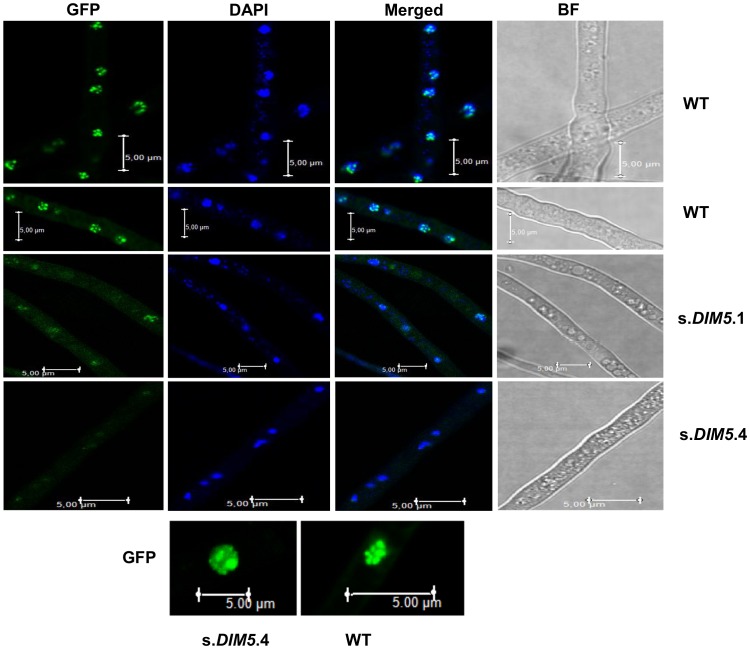
Location of LmHP1-GFP in nuclei of silenced-*LmDIM5* transformants and in the wild type isolate v23.1.3. The pBHt2-*LmHP1*-GFP plasmid was introduced in v23.1.3 and in two different silenced-*LmDIM5* transformants (.1 and .4, see Figure 3). Confocal laser scanning of fresh mycelium of v23.1.3 and of the two different silenced-*LmDIM5* transformants were observed. DNA was stained by DAPI, revealing the nucleus. One hundred nuclei were observed for the wild type strain or for the silenced-*LmDIM5*.1 and .4 transformants. Images are representative of all observations. Scale bars represent 5 µm.

We used LmHP1 as a convenient cytological marker for heterochromatin. As the nuclear localisation of HP1 depends on the action of *N. crassa* DIM-5 [30], we investigated localisation of LmHP1 in nuclei of wild type and in silenced-*LmDIM5* transformants. A LmHP1-GFP fusion under the control of the *LmHP1* endogenous promoter was introduced in the wild type strain and in two different silenced-*LmDIM5* transformants (.1 and .4, with residual *LmDIM5* expression of 20 and 16%, respectively). Nuclei were visualised by DAPI staining. In the wild type strain, LmHP1-GFP was located within strongly fluorescent foci throughout the nucleus and the same kind of dense foci were revealed by DAPI staining demonstrating coexistence of condensed and relatively open chromatin states along the chromosomes, and the preferential localisation of LmHP1-GFP to densely DAPI-stained chromatin regions (Figure 5). In the two silenced-*LmDIM5* transformants, DAPI staining in nuclei was more diffuse with fewer foci, and LmHP1-GFP was also seen as more diffuse staining throughout nuclei, with only the silenced-*LmDIM5.*1 showing a few clear foci in the nuclei (12 nuclei out of 100). The LmHP1-GFP localisation observed in the wild type strain and in the silenced-*LmDIM5* transformants is consistent with changes of HP1 localisation observed in *N. crassa dim-5* mutants [30]. Thus, LmHP1 localises in an LmDIM5-dependent manner to heterochromatic regions in *L. maculans*, and the silencing of *LmDIM5* triggers at least partial chromatin decondensation.

### Silencing of *LmHP1* and *LmDIM5* results in up-regulation of pathogenicity-related genes during mycelial growth

We compared expression of all *L. maculans* gene models during growth in axenic culture of a wild type isolate, the silenced-*LmDIM5*.4 transformant (with 16% residual expression of *LmDIM5*, and for which chromatin decondensation was more pronounced; Figure 5) and the silenced-*LmHP1*.5 transformant (with 35% residual expression of *LmHP1*) using oligoarrays. The expression level of eight or six percent of the *L.* maculans gene models were respectively influenced by the silencing of *LmDIM5* or *LmHP1* (978 or 746 out of 12,012 genes with a transcriptomic support). Regarding genes down-regulated compared to the wild type strain, 72% and 59% encode proteins with no homology or hypothetical proteins with unknown functions in respectively the silenced-*LmHP1* and the silenced-*LmDIM5* transformants. Among the genes encoding proteins with predicted functions, we identified respectively six and four percent of putative SSP-encoding genes (Tables S1 and S2). We also found a gene encoding a protein putatively related to pathogenesis down-regulated in the silenced-Lm*DIM5* transformant (Table S2). In the silenced-*LmDIM5* transformant, among the 390 up-regulated genes compared to the wild type isolate (Table S3), 283 encoded predicted proteins with no homology detected by BLAST or hypothetical proteins with unknown functions. The remaining up-regulated genes were mainly putative SSP-encoding genes, transporters and enzymes known to be accessory enzymes involved in the biosynthesis and transport of secondary metabolites (such as oxidases, MFS transporters, dehydrogenases and cytochrome P450 monooxygenases), CAZymes and the ortholog of the sterigmatocystin cluster regulatory gene in Aspergilli, *AflR* [38] (Table S3). Overall, the same functional categories of up-regulated genes were observed in the silenced-*LmHP1* background, where we found 369 up-regulated genes, including 206 genes with predicted functions (Table S4). In addition, we also identified seven genes involved in DNA repair, including three genes encoding DNA repair proteins of the Rad family (Table S4), suggesting that silencing of *LmHP1* may somehow trigger DNA damage. We further investigated the 71 genes that were up-regulated in both silenced-*LmDIM5* and -*LmHP1* transformants (Table S5). About half of these genes encode predicted or hypothetical proteins. Among genes encoding proteins with predicted functions most encode putative SSPs, cytochrome P450, transporters and CAZymes (Table S5). Notably, of the 31 highest up-regulated genes in both silenced-*LmDIM5* and -*Lm*HP1 transformants (fold-change>4; Table S5), 52% were putative SSP-encoding genes, while they only represent 5.1% of the genes in the genome [7]. This suggests a specific and strong effect of *LmHP1* and *LmDIM5* silencing on the expression of effector-encoding genes. We observed a stronger de-repression of gene expression in the silenced-*LmDIM5* transformant compared to the silenced-*LmHP1* transformant (Tables S3 and S4) that could result from the lower residual expression of *LmDIM5* compared to that of *LmHP1* in the transformants selected.

**Figure 6:**
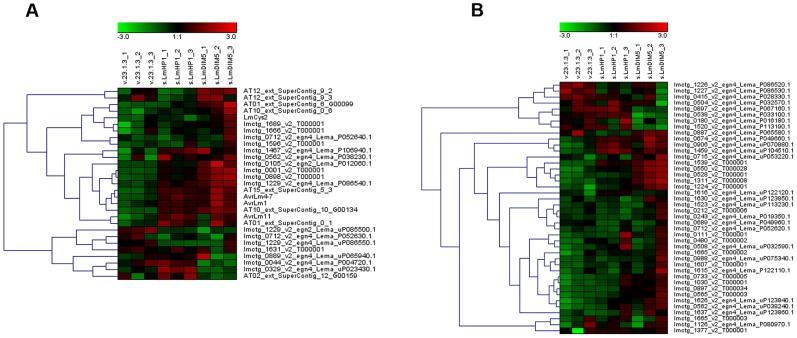
Expression of genes located in AT-isochores in the silenced-*LmHP1* and -*LmDIM5* transformants. Heat map showing genes that are up-regulated or down-regulated considering all Small Secreted Protein (SSP)-encoding genes (A) or non SSP-encoding genes (B) in AT-isochores in v23.1.3, in one silenced-*LmHP1* and in one silenced-*LmDIM5* transformants. Results are from three independent experiments (Exp. 1 to Exp. 3) for each comparison.

We next investigated the effect of *LmDIM5* and *LmHP1* silencing on the transcriptional behaviour of genes according to their genomic environment (Table 1). Whereas silencing of *LmDIM5* and *LmHP1* led to an up-regulation of ∼3% of genes located either in GC-isochores or in GC-equilibrated islands located in the middle of AT-isochores, 34% and 28% of genes located in AT-isochores were up-regulated in silenced-*LmDIM5* or -*LmHP1* backgrounds (Table 1). Remarkably, this effect was even more pronounced for the SSP-encoding genes as 41% of these genes, including *AvrLm1*, *AvrLm4-7* and *AvrLm11* were up-regulated when compared to the wild type strain (Table 1, Figures 6A and 6B). These observations support the idea that expression is regulated directly by alteration of H3K9me3 in AT-isochores of transformants in which *LmDIM5* and *LmHP1* are silenced. Moreover, genes located in AT-isochores that were up-regulated in both silenced-*LmHP1* and -*LmDIM5* backgrounds were all found up-regulated at 7 dpi during plant infection in the wild type isolate (Table 2). Taken together, these data support the idea that LmDIM5 and LmHP1 exert an epigenetic control on specific regions of the genome, and mainly on the AT-isochores that are expected to be associated with heterochromatic nucleosomes.

**Table 1:** Number of *L. maculans* genes up-regulated or down-regulated in a silenced-*LmHP1* or silenced-*LmDIM5* background according to their genomic location.

	GC-isochores		Borders between AT- and GC-isochores^a^		AT-isochores		GC-equilibrated islands^b^		All predicted genes
	SSPs	non SSPs	SSPs	non SSPs	SSPs	non SSPs	SSPs	non SSPs	
No. of genes present on the NimbleGen array with transcriptomic support	489	10,981	56	369	29	42	15	32	12,013
Genes overexpressed in silenced-*LmHP1* (%)^c^	3.9	2.8	8.9	3.5	41.4	19	6.6	0	3,1
Genes underexpressed in silenced-*LmHP1* (%)^c^	3.5	2.7	5.4	11.7	6.9	2.4	6.6	18.7	3.1
Genes overexpressed in silenced-*LmDIM5* (%)^c^	6.7	2.8	8.9	4.0	41	31	6.6	9.3	3.2
Genes underexpressed in silenced-*LmDIM5* (%)^c^	3.8	4.3	12.5	19.8	6.9	7.1	13.3	21.8	4.9

a Borders refer to 859 (±385) bp transition regions between AT-isochores and GC-isochores.

b GC-equilibrated islands in AT-isochores refer to regions of more than 1 kb within AT-isochores with a GC content >50%.

c Genes with more than 1.5-fold change in transcript level and an associated *p* value of <0.05 were considered as significantly differentially expressed in silenced-*LmHP1* or in silenced-*LmDIM5* transformants compared to the wild type (v23.1.3) isolate in axenic culture; expressed as a percent of genes with transcriptomic support.

**Table 2:** List of *L. maculans* genes located in AT-isochores and significantly up-regulated both in the silenced-*LmHP1* and silenced-*LmDIM5* transformants according to their transcriptional profiles during *in planta* infection.

SEQ_ID	Fold change in silenced-*LmHP1* a	Fold change in silenced-*LmDIM5* a	Function	Fold change 7 dpi^b^
lmctg_1626_v2_egn4_Lema_uP123840.1	1.63	2.24	predicted protein	7.38
AT10_ext_SuperContig_10_G00134	7.74	17.45	putative SSP-encoding gene	231.47
lmctg_1311_v2_T000008^c^	2.24	8.98	predicted protein	#N/A
lmctg_0674_v2_egn4_Lema_P049660.1	5.95	14.58	similar to AvrLm1 protein	327.73
AT15_ext_SuperContig_5_3	5.97	11.45	putative SSP-encoding gene	189.62
lmctg_1229_v2_egn4_Lema_P086540.1	3.00	8.04	similar to Six5	163.23
lmctg_1459_v2_egn4_Lema_uP104510.1	8.59	4.52	predicted protein	67.64
AvrLm1	5.60	16.93	AvrLm1	313.63
lmctg_1214_v2_egn4_Lema_P086290.1	8.80	16.83	AvrLm4-7	182.85
lmctg_1539_v2_T000001^c^	13.80	67.97	predicted protein	#N/A
lmctg_0562_v2_egn4_Lema_uP038240.1	1.77	2.14	predicted protein	3.99
lmctg_0550_v2_T000028^c^	1.85	4.69	predicted protein	#N/A
lmctg_0898_v2_T000001^c^	5.31	62.19	putative SSP-encoding gene	#N/A
lmctg_0001_v2_T000001^c^	6.65	77.16	putative SSP-encoding gene	#N/A
lmctg_0105_v2_egn2_Lema_P012060.1	2.97	30.18	putative SSP-encoding gene	1.94

a Genes with more than 1.5-fold change in transcript level and an associated *p* value of <0.05 were considered as significantly up-regulated in silenced-*LmHP1* or in silenced-*LmDIM5* transformants compared to the wild type (v23.1.3 isolate) in axenic culture.

b Fold-change of gene expression at 7 dpi relative to gene expression during mycelium growth (data from [7]).

c Genes suggested by new annotation.

**Figure 7:**
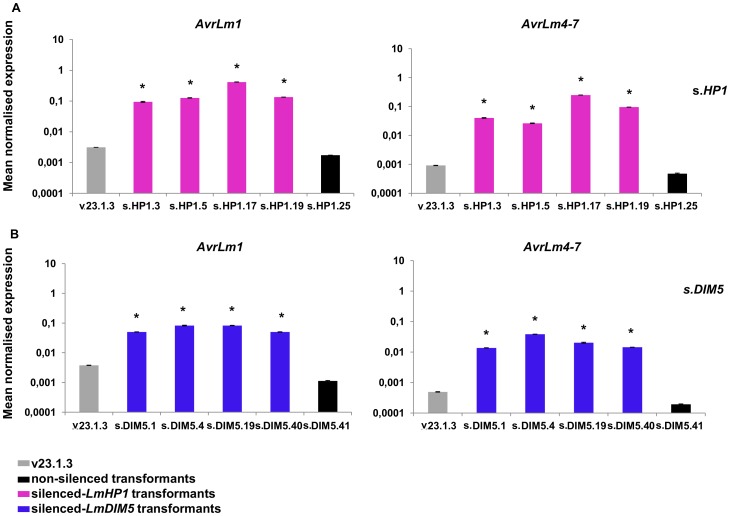
Expression of two effector genes in axenic culture in the silenced-*LmHP1* and -*LmDIM5* transformants. Quantitative RT-PCR analyses of expression were done in the wild type strain, in a transformant that has not been silenced either for *LmHP1* or *LmDIM5* and in the four selected transformants silenced for *LmHP1* or *LmDIM5* (Figure 3). (A) Quantification of *AvrLm1* and *AvrLm4-7* in the four silenced-*LmHP1* transformants or (B) in the four silenced-*LmDIM5* transformants compared to v23.1.3 (grey bar) and to a transformant that has not been silenced (black bars). Gene expression levels are relative to 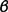 *-Lmtubulin*. Expression of *Lmactin* relative to 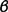 *-Lmtubulin* was used as control. Error bars indicate the standard deviation of two biological and technical repeats. Probability: * indicates *P*<0.05.

We analysed the expression of three effector genes located in AT-isochores (*AvrLm1*, *AvrLm4-7*, *LmCys2*) and one effector gene located in a GC-equilibrated island (*LmCys1*; I. Fudal and B. Profotova, unpublished data) by qRT-PCR during mycelial growth. Consistent with results from our oligoarray study, we observed a strong overexpression of *AvrLm1* and *AvrLm4-7* in the silenced-*LmHP1* (∼80- and 148-fold respectively; Figure 7A) and -*LmDIM5* transformants (∼25- and 63-fold respectively; Figure 7B), and less overexpression for *LmCys2* (∼10- and 2-fold in the silenced-*LmHP1* and -*LmDIM5*, respectively; data not shown). In contrast, *LmCys1*, the gene located in a GC-equilibrated island, was repressed (∼0.25- and 0.1-fold expression in the silenced-*LmHP1* and -*LmDIM5*, respectively; Tables S1 and S2). Thus, we found derepression of *LmCys2* in both silenced-*LmHP1* and -*LmDIM5* by qRT-PCR while no derepression was found using oligoarrays. These results confirm the oligoarray data but also suggest that oligoarrays likely underestimate the number of genes affected by the silencing, as well as the magnitude of derepression.

### Induced gene expression is correlated with a reduction of H3K9 trimethylation at *AvrLm1* and *AvrLm4-7* loci in axenic culture

**Figure 8:**
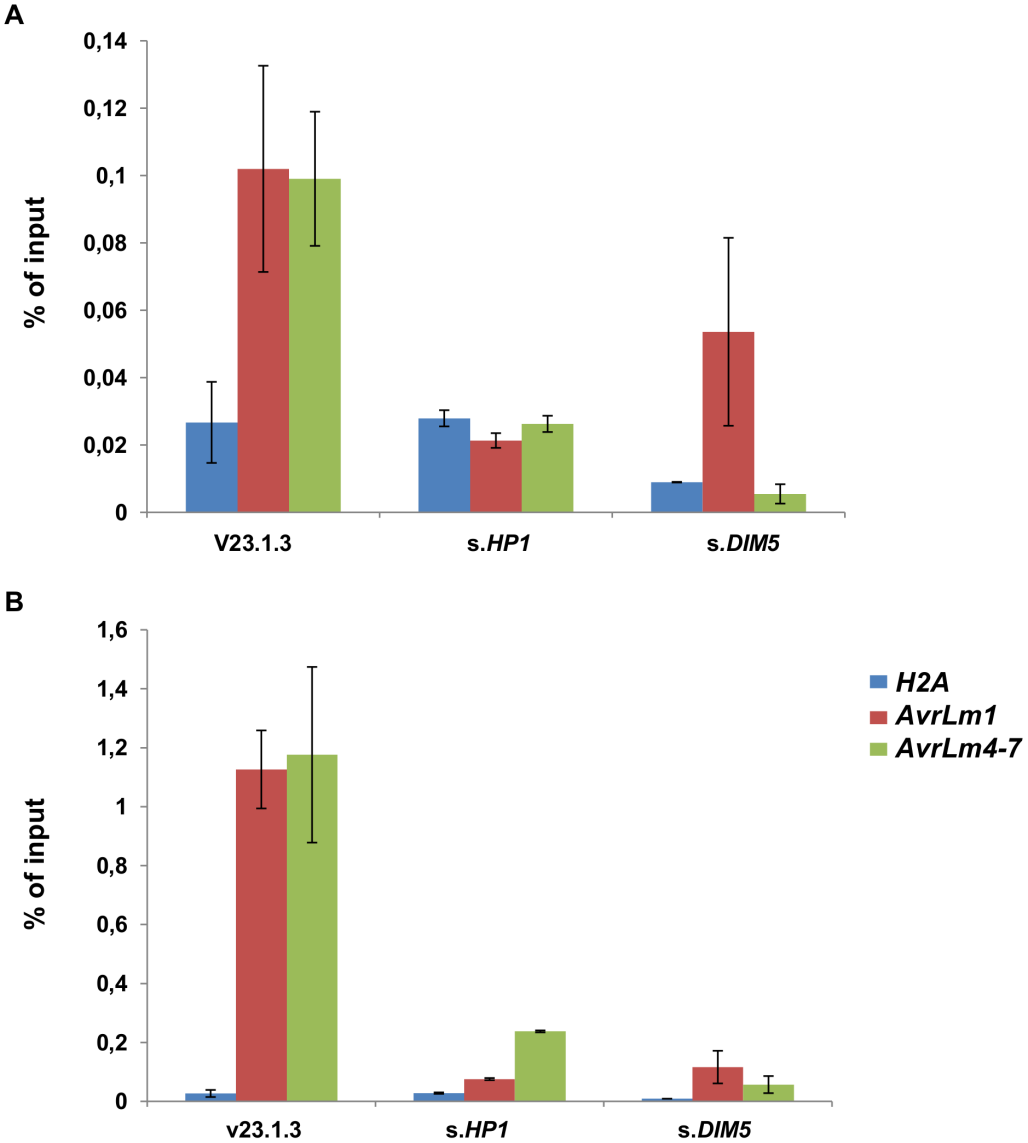
Trimethylation of lysine 9 of histone H3 (H3K9me3) is reduced in silenced-*LmHP1* and silenced*-LmDIM5* backgrounds. Chromatin immunoprecipitation analysis was performed to assess changes in the heterochromatic H3K9me3 mark in (A) the coding regions or (B) the promoters of *AvrLm1* and *AvrLm4-7*. We used portions of the histone *H2A* gene as a negative control of H3K9me3 enrichment. Data were normalised using the “percent of input” method. *AvrLm1*_Pro and *AvrLm4-7*_Pro: promoter regions of *AvrLm1* or *AvrLm4-7*. Error bars indicate the standard deviation of two biological and technical repeats.

To investigate whether induction of effector gene expression in axenic culture after silencing of *LmDIM5* or *LmHP1* was associated with changes of the H3K9me3 level, we performed chromatin immunoprecipitation (ChIP) experiments on strain v23.1.3, and the silenced-*LmHP1*.5 and -*LmDIM5*.4 transformants. We assayed *AvrLm1* and *AvrLm4-7* coding regions, their promoters and used the histone *H2A* gene as a control. To precipitate nucleosomes associated with H3K9me3, we used an antibody that had been used successfully in studies with *N. crassa* [39], [40], *Trichoderma reesei* [41], [42], *Fusarium fujikuroi* [43] and *Fusarium graminearum* [44]. In the wild type isolate, we observed higher levels of H3K9me3 in the promoters of *AvrLm1* and *AvrLm4-*7 compared to their coding regions (Figures 8A and 8B). H3K9me3 levels were strongly reduced in *AvrLm1* and *AvrLm4-7* for all regions tested in both the silenced-*LmHP1* and -*LmDIM5* transformants when compared to the wild type strain (Figures 8A and 8B); as expected, silencing of *LmDIM5* or *LmHP1* had no effect on the low H3K9me3 level in the *H2A* gene. Thus, overexpression of at least *AvrLm1* and *AvrLm4-7* observed in axenic culture in the silenced-*LmHP1* and -*LmDIM5* transformants is correlated to a measurable decrease in H3K9me3 levels in these genomic regions. Precipitations with H3K4me2 antibody were done in parallel as controls for ChIP efficiency (Figure S1). This modification has been described as associated with active regions of chromatin [45]. Accordingly, levels of H3K4me2 in the constitutively expressed gene, *H2A*, are high either in the WT strain or in both silenced-*LmHP1* and -*LmDIM5* transformants. In the WT strain, levels of H3K4me2 are reduced by 12 to 614 times in the coding sequences and promoters of *AvrLm1* or *AvrLm4-7* compared to the coding sequence of H2A (Figure S1), consistently with a repression of effector gene during axenic culture. However, in *L. maculans*, and based on levels of H3K4me2 observed in the silenced-*LmDIM5* and -*LmHP1* transformants, no correlation seems to exist between induction of effector gene expression and a modification in H3K4me2 levels (Figure S1).

### Silencing of *LmHP1* or *LmDIM5* does not alter the pattern of effector gene expression during primary infection

**Figure 9:**
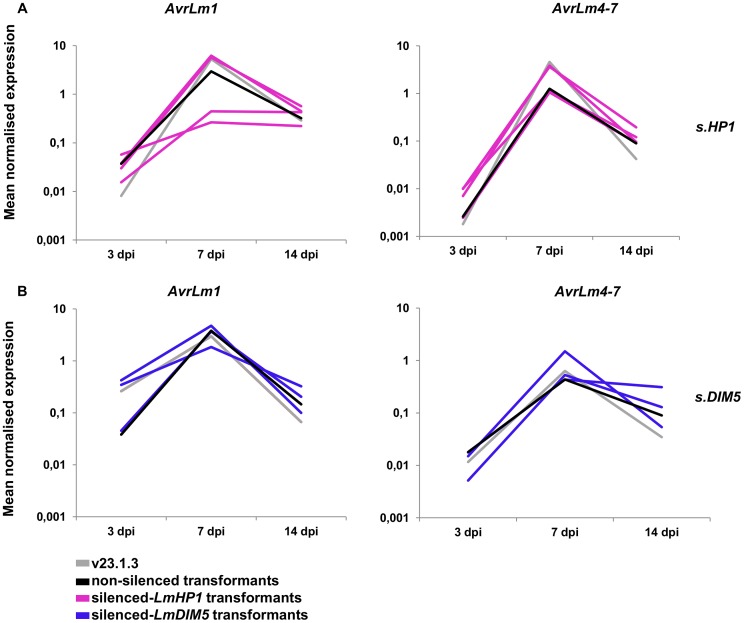
Expression of two effector genes in the silenced-*LmHP1* and -*LmDIM5* transformants during infection. Expression of *AvrLm1* and *AvrLm4-7* was measured by qRT-PCR from 3 to 14 days post infection (dpi) of oilseed rape cotyledons (susceptible cultivar Westar) in a wild type strain (grey lines), a transformant that has not been silenced either for *LmHP1* or *LmDIM5* (black lines) and (A) four silenced-*LmHP1* transformants or (B) four silenced*-LmDIM5* transformants by qRT-PCR. Gene expression levels are relative to *β-Lmtubulin*. Expression of *Lmactin* relative to *β-Lmtubulin* was used as a control.

Expression of *AvrLm1*, *AvrLm4-7*, *LmCys1* and *LmCys2* was monitored during oilseed rape infection (3, 7 and 14 dpi) by qRT-PCR in the silenced-*LmHP1* and -*LmDIM5* transformants (Figures 9A and 9B; data not shown) and compared to the wild type strain or to non-silenced transformants. Expression profiles were similar in all isolates, with a peak of expression at 7 dpi. Thus, silencing of either *LmHP1* or *LmDIM5* did not alter the profiles of effector gene expression during primary infection.

### Genomic environment of effector genes has an impact on their expression level

**Figure 10:**
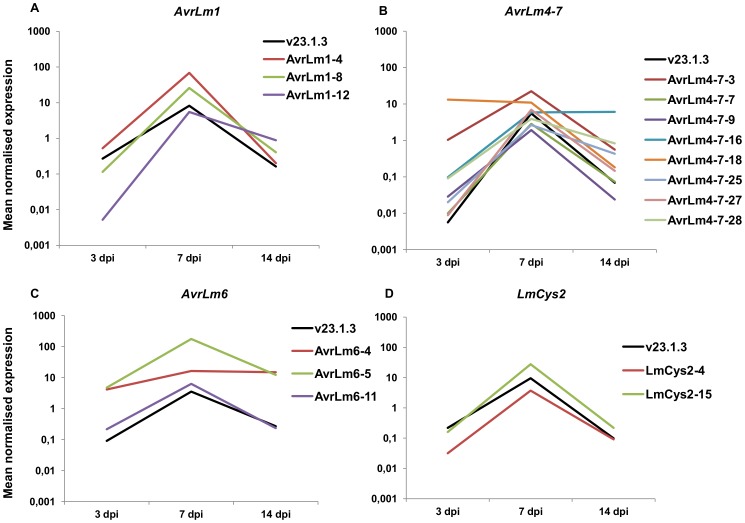
Expression of four effector genes *in planta* following ectopic integration in GC-isochores of the genome. Expression of the effector genes ***AvrLm1*** **(A),** ***AvrLm4-7*** **(B),** ***AvrLm6*** **(C) and** ***LmCys2*** **(D)** was analysed by qRT-PCR in the wild type isolate v23.1.3, in which these genes are located in AT-isochores and compared to their expression in different transformants (v29.3.1-AvrLm1-x, NzT4-AvrLm4-7-x, v29.3.1-AvrLm6-x, or v29.3.1-LmCys2-x), in which they are inserted in GC-isochores. Gene expression levels are relative to *β-Lmtubulin*. 3 to 14 dpi: RT-PCR products obtained from RNA isolated from oilseed rape cotyledons (susceptible cultivar Westar) 3 to 14 days post infection. RNA extracted from uninfected cotyledons and water were used as negative controls. Each data point is the average of two technical repeats (two RT-PCRs).

As previously described, *Agrobacterium tumefaciens*-mediated transformation (ATMT) of *L. maculans* mainly results in integration of transgenes in GC-isochores [46]. Isolates naturally lacking the effector genes *AvrLm1*, *AvrLm6*, *AvrLm4-7* and *LmCys2* were subjected to ATMT complementation [19]–[21]. Thermal Asymmetric InterLaced (TAIL)-PCRs were performed on 66 transformants in order to check that the integrated T-DNA was indeed located into GC-isochores. T-DNA flanking sequences were recovered for 54% of the transformants (Table S6) and were identified in the *L. maculans* genome using BLAST. After screening, 16 transformants with identified T-DNA sequences were kept for further study (24% of the sequences; see Table S6 for details), corresponding to insertions in GC-isochores of 12 different supercontigs; ten were inserted into coding sequences, two into promoters and four into intergenic regions (see Table S7 for details on insertions). In all cases, expression of *AvrLm1*, *AvrLm6*, *LmCys2* and *AvrLm4-7* in axenic culture was greatly increased, varying from 8- to 1270-fold compared to that of the wild type isolate (Table 3). Expression profiles *in planta* were similar to that of the wild type isolate with a peak of expression at 7 dpi (Figure 10), except for transformants NzT4-*AvrLm4-7*-16 and -18. These data corroborated the effect of *LmHP1* and *LmDIM5* silencing, which led to overexpression of effector genes in axenic culture with no altered expression pattern during primary infection. Our data strongly suggest that chromatin structure of AT-isochores represses expression of effector genes embedded in these regions, at least during growth in axenic culture. In contrast, during primary leaf infection, expression of effector genes located in AT-isochores did not depend on the chromatin context alone.

**Table 3:** Expression of *AvrLm1*, *AvrLm6*, *AvrLm4-7* and *LmCys2* in *axenic culture* of mycelium following ectopic integration in GC-isochores of the genome.

Isolate/Transformant^a^	Mean normalised expression during *in vitro* growth^b^	Ratio of expression transformant/WT
*LmCys2* expression		
v23.1.3	2.84E-04	
v29.3.1-*LmCys2*-4	3.96E-03	13.94
v29.3.1-*LmCys2*-15	1.19E-02	41.87
*AvrLm4-7* expression		
V23.1.3	2.36E-04	
NzT4-*AvrLm4-7*-3	4.85E-02	205.69
NzT4-*AvrLm4-7*-7	1.86E-02	78.81
NzT4-*AvrLm4-7*-9	7.23E-03	30.7
NzT4-*AvrLm4-7*-16	2.43E-02	103.05
NzT4-*AvrLm4-7*-18	1.66E-02	70.55
NzT4-*AvrLm4-7*-25	6.61E-02	280.66
NzT4-*AvrLm4-7*-27	7.49E-03	31.81
NzT4-*AvrLm4-7*-28	1.01E-01	429.97
*AvrLm6* expression		
v23.1.3	1.15E-04	
v29.3.1-*AvrLm6*-4	1.47E-01	1269.81
v29.3.1-*AvrLm6*-5	3.55E-03	30.75
v29.3.1-*AvrLm6*-11	1.70E-03	14.71
*AvrLm1* expression		
v23.1.3	1.44E-03	
v29.3.1-*AvrLm1*-8	1.37E+00	955.83
v29.3.1-*AvrLm1*-4	1.12E-02	7.79
v29.3.1-*AvrLm1*-12	4.88E-02	33.93

a *AvrLm1*, *AvrLm6*, *AvrLm4-7* and *LmCys2* are in AT-isochores in the wild type (v23.1.3) isolate but inserted in GC-isochores in transformants (v29.3.1-*AvrLm1*-x, v29.3.1-*AvrLm6*-x, NzT4-*AvrLm4-7*-x or v29.3.1-*LmCys2*-x). Their expression was measured by qRT-PCR.

b Gene expression levels are relative to *β-Lmtubulin* and calculated as described [94]. Each value is the average of two biological replicates (two extractions from different biological material) and two technical replicates (two RT-PCRs).

## Discussion

AT-isochores of the *L. maculans* genome were found to be enriched in effector genes sharing common expression profiles, with repression of expression during mycelial growth but drastic induction during the early stages of oilseed rape leaf infection [7]. We recently showed that the location of effector genes in this genomic environment has selective advantages by allowing extremely rapid responses to resistance gene selection generated by mutation and/or recombination [47] as it was also reported for *Phytophthora infestans* [17]. Here, we show that the genomic environment is important for control of gene regulation, and thus may confer a second adaptive advantage as it allows repression of genes specifically needed for pathogenicity during vegetative growth and possibly allows rapid induction of genes required for infection. To elucidate the mechanism involved, we investigated possible chromatin-mediated epigenetic repression through histone H3K9 trimethylation in strains where key players involved in heterochromatin formation (*LmHP1* and *LmDIM5*) were silenced by RNAi.

### Roles of LmHP1 and LmDIM5 in *L. maculans*

The domain structures of HP1 and DIM-5 proteins and their functions are well conserved in various eukaryotes, from plants to mammals. The homologue of the *N. crassa* histone H3 methyltransferase DIM-5 identified in *L. maculans* revealed functional domains typical of Suvar39/Clr4/DIM-5 orthologs. Notably, nine cysteines shaping the zinc cluster of the pre-SET domain [35], as well as three residues (N, H, Y) with catalytic roles are well conserved [48] (Figure 2). Consistent with data obtained from *N. crassa* [28] and *Aspergillus fumigatus* [49], the silenced-*LmDIM5* transformants of *L. maculans* showed growth defects. In contrast to *DIM-5* mutants of *A. fumigatus* [49], silenced-*LmDIM5* isolates showed pathogenicity defects. HP1 is an adapter protein that recognises and binds H3K9 trimethylation [50], though the closest *Arabidopsis thaliana* homologue, LHP1, also binds histone H3 lysine 27 methylation (H3K27me3) [51]–[53]. In *N. crassa*, HP1 recruits the DNA methyltransferase DIM-2, and has been shown to be essential for all DNA methylation in heterochromatic regions [30]–[32]. The HP1 protein of *L. maculans* revealed functional domains typical of all HP1 orthologs. Notably, the three aromatic residues of the chromo domain, forming a binding pocket for the N-methyl groups of the H3 tail [54] are conserved from *N. crassa* to *H. sapiens* (Figure 1). Besides heterochromatin formation, multiple roles are attributed to HP1, including involvement in centromere maintenance, genome integrity, sister-chromatin cohesion, and DNA repair [40], [55]–[57], but defects observed depend on the organism studied to date. Growth defects are observed in all *HP1* mutants of *N. crassa* [30], and we found here similar defects in transformants in which *LmHP1* was only partially silenced by RNAi. No such growth defects have been reported in *HP1* mutans of *F. graminearum* or *A. nidulans* [58], [59]. *HP1* mutations trigger larval death in *D. melanogaster* [60], [61]. In *L. maculans*, we observed that only one-third of primary *LmHP1* transformants could be recovered following the first step of selection. In addition, the residual expression of *LmHP1* in silenced isolates remained high (always more than 30% of the control), in contrast to what we observed for the silenced-*LmDIM5* transformants. These data suggest that, as for *D. melanogaster*, *LmHP1* deletion may be lethal. In contrast to transformants with silenced *LmDIM5*, transformants with silenced *LmHP1* showed no pathogenicity defects. Whether maintenance of pathogenicity of silenced-*LmHP1* transformants is due to the lack of drastic silencing of *LmHP1* is an open question, as the involvement of HP1 in pathogenicity has not been analysed in any other plant pathogen. The silencing of *LmDIM5* was associated with a decrease in pathogenicity ability that did not depend on the level of effector gene expression during infection. As *LmHP1* and *LmDIM5* silencing resulted in a deregulation of expression of a considerable number of genes, we can assume that the pathogenicity defect could also be linked to another physiological defect that would be important for infection processes that would be affected. As HP1 localisation in *N. crassa* relies on H3K9me3, catalysed by DIM-5, we investigated LmHP1 localisation in the wild type strain and in silenced-*LmDIM5* transformants. As in *N. crassa*, LmHP1 location in *L. maculans* relies on proper function of LmDIM5, and there seems to be at least some defect in chromatin structure in the silenced-*LmDIM5* background. Our ChIP experiments showed that *LmHP1* and *LmDIM5* are responsible for generating heterochromatic regions at two *L. maculans* avirulence gene loci, and that induction of effector gene expression in axenic cultures in either silenced-*LmHP1* or -*LmDIM5* transformants relied on reduction of H3K9me3 levels in these regions. Interestingly, the level of H3K9me3 is slightly higher in promoters of *AvrLm1* and *AvrLm4-7* compared to the coding regions, suggesting that promoters are the preferred targets for epigenetic regulation of gene expression. Overall, our native ChIP experiments tend to support the idea that AT-isochores with effector genes represent epigenetically controlled facultative heterochromatin. The reduction of H3K9me3 in a silenced-*LmDIM5* background is consistent with DIM-5 function. We also showed here that levels of H3K9me3 is reduced in coding sequences and promoters of *AvrLm1* and *AvrLm4-7* while HP1 acts downstream of DIM-5. In *S. pombe*, the HP1 ortholog Swi6, through self-association, is required for interaction with H3K9me3 but also for the recruitment of the DIM-5 ortholog, Clr4 [62] which is in accordance with the reduction of H3K9me3 levels observed here in a silenced-L*mHP1* background. Regarding H3K4me2, our results suggest that this modification does not replace H3K9me3 when genes located in facultative heterochromatin are actively transcribed. Such behaviour for this modification was also observed in *F. graminearum* [44].

In our oligoarray analyses, in addition to genes located in AT-isochores largely affected in both the silenced-*LmHP1* and the silenced-*LmDIM5* transformants, we also found 49 genes located in GC-isochores up-regulated, such as genes encoding for nine SSPs and a cytochrome P450 protein. This suggests that hotspots in GC-isochores may also be subject to chromatin-based regulation; alternatively, this up-regulation may be indirect and a consequence of expression of genes located in AT-isochores. Additional ChIP and ChIP-sequencing studies are underway to address this question. To our knowledge, up-regulation of numerous genes involved in DNA repair in transformants with silenced-*LmHP1* is a novel finding. Previous results suggest that some heterochromatin mutants, including *dim-5* but not *hpo* (*N. crassa HP1)* are mutagen sensitive [63]. Silencing or deletion of *HP1* may trigger DNA damage, which would explain pleiotropic effects that have been also observed in other organisms as previously mentioned.

### Pathogenicity-related genes are often under a double control

We show here that an epigenetic mechanism efficiently tunes effector gene expression and that by removal of H3K9me3, expression of these genes can be rapidly activated in response to changing environmental conditions. It is known that an intimate link sometimes exists between epigenetic regulation of gene expression and transcription factors, the latter triggering chromatin condensation or decondensation, and the three dimensional structure of the chromatin governs promoter access to regulatory proteins such as transcription factors. There is precedence for this idea, as transcription factors can be recruited to promote the reversion of epigenetic silencing, as for example, the BZLF1 transcription factor of the Epstein-Barr virus initiates virus synthesis [64]. In contrast, in *Plasmodium falciparum*, PfSIP2 initiates the formation of heterochromatin leading to epigenetic silencing of genes involved in pathogenesis [65]. Transcription factors are recruited after chromatin decondensation to specifically regulate certain genes; for example, expression of secondary metabolite gene clusters in Aspergilli requires modulation of H3K9me3 levels and action of AflR, a specific transcription factor [38], [66], [67], thus generating multi-layered control. Based on this, we could suspect that such a multiple control may regulate effector gene expression in *L. maculans*. Nevertheless, whether one, or several, transcription factor(s) is involved in the concerted expression of effector genes in *L. maculans* remains to be elucidated. A few transcriptional regulators involved in effector gene expression have already been identified, such as SGE1 of *F. oxysporum*, which is essential for parasitic growth and Fox1 of *U. maydis*, a forkhead transcription factor which is also required for pathogenic development into the plant [68], [69].

### Epigenetic-mediated control of pathogenicity-related gene expression: An efficient and universal mechanism?

While all the cells in an organism contain the same genetic information, epigenetic modifications enable a specialisation of function during development, stress or infection mechanisms. In 1942, Conrad Waddington named these different states “epigenotypes”, to indicate that conditions set up under one particular situation to switch gene expression on or off can efficiently and quickly adapt to a new situation, independently of the genotype [70]. If genetic mutations can help an organism to adapt and survive along evolution, the presumed epigenetic code provides a more sensitive and rapid way to respond to environmental stress than DNA sequences themselves. For example, in viruses like human Herpes Simplex Virus 1 and 2, epigenetic modifications determine either latency of this virus, *i.e.* its quiescent state when there is no *de novo* production of the virus or lytic infection, *i.e.* its active state when a large number of viral genes are expressed leading to cell death [71]. As mentioned above, effector genes show common features, notably they remain globally silenced during mycelial growth and are strongly up-regulated upon infection. Producing effectors implies an energy cost for the organism. Thus, it seems plausible that effector production is tightly regulated to avoid wasting energy under conditions when the gene products are not needed. Epigenetic repression is lifted at the precise stage where effectors are required, allowing for finely tuned regulation.

The work presented here is, to our knowledge, the first report of chromatin-mediated epigenetic control of fungal effector gene expression. In organisms as phylogenetically distant and diverse as Apicomplexa, Euglenozoa, Eozoa or Ascomycota (including *Plasmodium* spp., *Trypanosoma* spp., *Leishmania* spp., *Giardia lamblia* and *Candida* spp.), families of genes involved in pathogenicity are frequently located in subtelomeric regions and are variantly expressed, to escape the host immune system, through an epigenetic control [72]–[74].

In fungi, particularly Aspergilli, epigenetic control is involved in the regulation of secondary metabolite genes. Genes involved in secondary metabolites production are often organised in clusters [75], including their respective transcriptional activators (*e.g.* AflR for aflatoxin and sterigmatocystin production; [38], [66], [67]). Clusters are often near the chromosome ends (“subtelomeric”), which may allow for regional regulation mediated by chromatin modifications. These modifications appear to be controlled by a global regulator, LaeA [76], a putative histone methyltransferase, as well as HP1 and DIM-5 homologues [59], [77]. A similar system has been described in *F. graminearum* [58]. In *L. maculans*, we found that LmHP1 modulates the expression of three polyketide synthase and nonribosomal peptide synthetase genes, and both LmHP1 and LmDIM5 influence transcription of genes encoding accessory enzymes involved in the modifications of secondary metabolites.

To date, *L. maculans* is the only fungus for which an isochore-like structure of the genome has been published [7] but there is evidence for the existence of this genome structure from other fungi. In the hemibiotrophic dothideomycete *Venturia inaequalis*, the region containing the avirulence gene *AvrVg* is located in isochores with widely different GC content, which may be consistent with an isochore-like genome structure [78]. This organisation is also identifiable in the genomes of two additional dothideomycetes, *Mycosphaerella fijiensis* (http://genome.jgi.doe.gov/Mycfi2/Mycfi2.home.html) and *Cladosporium fulvum*, which contain effector-encoding genes in repeat-rich regions, which were presumably mutated by RIP [79]. Thus, similar epigenetic mechanisms to control expression of genes located in these highly dynamic regions may occur in these organisms. Moreover, the location of effector genes in subtelomeric regions, regions enriched in repeats or on CDC is a common feature of plant pathogenic fungi. Like *Nectria haematococca* [80] and *F. oxysporum* [16], *L. maculans* genome contains a CDC, invaded by repeats. This CDC harbours an effector gene, *AvrLm11* [14], which is up-regulated in axenic culture in a silenced-*LmHP1* background. This suggests that genes located on supernumerary chromosomes in *N. haematococca* or *F. oxysporum* may be likewise regulated. In combination, these data strongly suggest that in the course of evolution some epigenetic mechanisms to repress and control expression of genes involved in pathogenicity have been conserved across kingdoms.

### Switch from repression to expression: What could be the signal?

Mechanisms controlling gene activation or repression at the chromatin level are under intense scrutiny but signals triggering the switch between two chromatin states remain largely unknown. Here, we show that effector genes are repressed under axenic conditions and that repression is lifted upon leaf infection. This suggests that a particular signal is recognised by *L. maculans* to abolish epigenetic silencing of pathogenicity-related genes. While signals produced by plant roots begin to be characterised [81], nothing is known about the plant signals produced by leaves that could induce effector gene expression. As mentioned above, production of fungal secondary metabolites is under both general chromatin-mediated as well as specific transcription factor-mediated control, both of which likely respond to various environmental factors, for example nitrogen [43], [82]. Considering the induction of effector gene expression, the *Avr9* gene is the only effector gene induced upon nitrogen starvation in *C. fulvum* [83] but so far no effects of nitrogen levels on the expression of effector genes in *L. maculans* [19] or other fungi have been found. Identification of environmental factors triggering effector gene expression as well as plant genotypes that will be less favourable for the expression of effector genes is thus a promising field for research to prevent or limit disease development.

## Materials and Methods

### Fungal isolates

The isolates v23.1.3 (*AvrLm1-AvrLm4-AvrLm6-AvrLm7*) and v29.3.1 (*avrLm1*-*AvrLm4-avrLm6-AvrLm7*), NzT4 (*AvrLm1-avrLm4-avrLm6-avrLm7*) of *L. maculans* [84] were used as hosts for genetic transformations. Transformants used here to assess effect of genomic context on effector gene expression were previously described [19]–[21] and our unpublished data, and generated during the positional cloning of *AvrLm1*, *AvrLm6* and *AvrLm4-7*: v29.3.1-*AvrLm1*, v29.3.1-*AvrLm6*, v29.3.1-*LmCys2* and NzT4-*AvrLm4-7*. v29.3.1-*AvrLm1*, v29.3.1-*AvrLm6* and v29.3.1-*LmCys2* transformants corresponded to v29.3.1 isolates in which *AvrLm1*, *AvrLm6* or *LmCys2* allele of v23.1.3 were introduced using vector pBBH [19] or pBHt2 [85] (11, 13 and 15 transformants, respectively, were tested). NzT4-*AvrLm4-7* transformants corresponded to NzT4 isolate in which the *AvrLm4-7* allele of v23.1.3 was introduced using vector pPZPnat1 [86] (27 transformants tested). Fungal cultures, sporulating cultures and conidia production were maintained or collected as previously described [87]. For chromatin immunoprecipitation, tissue was grown on V8 agar medium at room temperature in the dark for 10 days. Mycelium was inoculated into 75 ml of Fries liquid medium in 250 ml Erlenmeyer flasks. Tissue was harvested after growing for 7 to 12 days in the dark at 27°C.

### Pathogenicity and growth of isolates

Pathogenicity assays were performed as described [88] on cotyledons of 15-day-old plantlets of cultivar Westar, a highly susceptible cultivar of *Brassica napus*. Plants were incubated in a growth chamber at 16/24°C (night/day) with a 12 h photoperiod. Symptoms were scored on 10–12 plants, at 14 days after inoculation using the IMASCORE rating scale comprising six infection classes (IC), where IC1 to IC3 correspond to various level of resistance of the plant and IC4 to IC6 to full susceptibility [89], with two biological replicates. Growth assays were performed by deposition of a 5-mm plug at the centre of 90-mm Petri dishes (containing 25 ml of V8 juice agar medium). Radial growth was measured at 10 days after incubation in a growth chamber, with three biological replicates.

### Fungal crosses

Assessment of sexual reproduction of silenced-*LmHP1* and silenced-*LmDIM5* transformants compared to non silenced transformants was performed by crossing with the v24.1.2 strain, a near isogenic isolate of v23.1.3, but of opposite mating type [90]. Crosses of v23.1.3 with v24.1.2 were performed as a control.

### DNA and RNA manipulation

For PCR, genomic DNA was extracted from conidia with the DNAeasy 96 plant Kit (Qiagen S.A., Courtaboeuf, France) and PCR amplifications were done as previously described [20]. Sequencing was performed using a Beckman Coulter CEQ 8000 automated sequencer (Beckman Coulter, CA, U.S.A) according to the manufacturer's instruction. Procedures for gel electrophoresis have been reported [91] and were adapted from procedures described by Sambrook and Russell [92]. Total RNA was extracted from mycelium grown for one week in Fries liquid medium, and from infected leaf tissues as previously described [20].

### Native Chromatin ImmunoPrecipitation (N-ChIP)

We first attempted to adapt the ChIP protocol developed for *N. crassa* and *Fusarium* species [28], [43], but found that sonication or bead-beating and formaldehyde crosslinking resulted in very poor shearing or yields of precipitated DNA. We thus subjected *L. maculans* to “native ChIP” (no crosslinking) and isolated mono- and dinucleosomes after digesting ∼300 mg of mycelium per sample with microccocal nuclease (MNase) for 25 min at 37°C (L.R. Connolly and M. Freitag, unpublished data). Input (40 µl of the whole cell lysate) was stored (−20°C) for each sample and used to normalise data from qPCR and qualitative PCR. For immunoprecipitation, each sample was split into two replicates of 250 µl lysate each and 3.5 µl H3K9me3 antibody (Active Motif 39161) was added for a total of two replicates per sample. Precipitations with H3K4me2 antibody (Millipore 07-030) were done in parallel as controls for ChIP efficiency. Because we used a native ChIP protocol, yields were consistently lower than from ChIP experiments with crosslinked chromatin.

### TAIL-PCR experiments

To recover DNA sequences flanking *AvrLm1*, *AvrLm6*, *LmCys2* or *AvrLm4-7* in transformants v29.3.1-*AvrLm1*, v29.3.1-*AvrLm6*, v29.3.1-*LmCys2* and NzT4-*AvrLm4-7* respectively, TAIL-PCR [85], [93] was used. pPZPnat1 sequence-specific primers were designed to match primer characteristics as described [85] (Table S8). For pBBH and pBHt2 vectors, primers used were as designed previously [85], [88] (Table S8). Arbitrary Degenerated (AD) primers used in association with the vector-specific TAIL-PCR primers were identical to primer AD2 [93]. For v29.3.1-*AvrLm1* transformants, TAIL-PCR was performed as described [93], while for v29.3.1-*AvrLm6*, v29.3.1-*LmCys2* and NzT4-*AvrLm4-7* transformants, TAIL-PCR was performed as described [85]. Tertiary TAIL-PCR products were purified using the Nucleospin Extract II purification Kit (Macherey-Nagel, Hoerd, France) and were used as template for DNA sequencing using the specific tertiary border primer (LB3) as a sequencing primer (Table S8).

### qRT-PCR

Quantitative RT-PCR was performed using a model 7900 real-time PCR machine (Applied Biosystems) and Absolute SYBR Green ROX dUTP Mix (ABgene, Courtaboeuf, France) as previously described [20]. For each condition tested, two different RNA extractions from two different biological samples and two reverse transcriptions for each biological repeat were performed. Primers used for qRT-PCR are described in Table S8. Ct values were analysed as described [94] for analyses of expression profiles. *β -Lmtubulin* was used as a constitutively expressed reference gene. Expression of *Lmactin* relative to *β-Lmtubulin* was used as control.

For qPCR on ChIP DNA, primers were designed to amplify products between 50 to 150 bp (Table S8). For each reaction, we used 1 µl of the sample and performed reaction in duplicates. The “percent of input” method was used to calculate the immunoprecipitated fraction for each primer pair according to the following formula: %of input = 100×(1+E)∧(Ct_input_−Ct_bound_), where E is the primer efficiency of each primer pair designed to amplify the amplicon, Ct input is the Ct of the fraction recovered after digestion by MNase and Ct bound is the Ct of the immunoprecipitated sample.

### Whole-genome oligoarray analyses of expression of *L. maculans* genes

To compare expression of effector genes located in AT- and GC-isochores, oligoarray data previously obtained [7] and deposited in the Gene Expression Omnibus (GEO) under accession code GSE27152 were used. Additional *L. maculans* whole-genome expression arrays were manufactured by NimbleGen Systems Limited (Madison, WI) in order to compare whole genome expression in mycelium of wild type isolate v23.1.3 and transformants silenced for *LmHP1* or *LmDIM5*. These arrays contain 5 independent, non-identical, 60-mer probes per gene model, each duplicated on the array. Gene models included were 12,457 EuGene-predicted gene models and 467 additional genes, 2,008 random 60-mer control probes and labelling controls. The oligoarray data are available from the NCBI GEO under the accession number GSE50616. Total RNA was extracted using TRIzol reagent (Invitrogen) according to the manufacturer's protocol from mycelium grown for one week in Fries liquid medium. Total RNA was treated with RNase-Free DNase I (New England Biolabs). Total RNA preparations (three biological replicates for each sample) were amplified by PartnerChip (Evry, France) using the SMART PCR cDNA Synthesis Kit (Invitrogen) according to the manufacturer's instructions. Single dye labeling of samples, hybridisation procedures, data acquisition, background correction and normalisation were performed at the PartnerChip facilities following the standard protocol defined by NimbleGen [95], [96]. Average expression levels were calculated for each gene from the independent probes on the array and were further analysed.

Gene-normalised data were subjected to Analysis of NimbleGen Array Interface Suite (ANAIS; http://anais.versailles.inra.fr; [97]). ANAIS performs an ANOVA test on log_10_ transformed data to identify statistically differentially expressed genes. This test uses the observed variance of gene measurements across the three replicated experiments. To account for multiple tests, the ANOVA *p* values are further subjected to Bonferroni correction. Transcripts with *p* values<0.05 and >1.5-fold change in transcript levels were considered as significantly differentially expressed in the silenced-*LmHP1* or in the silenced-*LmDIM5* compared to the wild type strain v23.1.3. To estimate the signal-to-noise threshold (signal background), ANAIS calculates the median of the intensity of all of the random probes present on the oligoarray, and provides adjustable cut-off levels relative to that value. Gene models with an expression higher than three-times the median of random probe intensities in at least two of three biological replicates were considered as transcribed.

### Vector construction and fungal transformation

Vectors pPZPnat1-*LmHP1* and pPZPnat1-*LmDIM5* for RNAi-mediated silencing of *LmHP1* and *LmDIM5* genes were constructed as described [98] with some modifications [20]. pJK11 vector contains a *Glomerella cingulata gpd*A promoter fragment and an *A. nidulans trp*C terminator fragment, separated by a multiple locus site where inverted repeats of coding sequence of *LmHP1* and *LmDIM5* have been cloned using the primers described in Table S8. The *LmHP1* sense fragment of the inverted repeat was amplified from cDNA of v23.1.3 mycelium using primers Silent*LmHP1-Hind*III*+* and Silent*LmHP1-Bam*HI− and digested with *Hind*III and *Bam*HI. The antisense region was amplified with primers Silent*LmHP1-Bam*HI*+* and Silent*LmHP1-Eco*RI- and digested with *Bam*HI and *Eco*RI. Sense and antisense fragments were ligated into an *Eco*RI-*Hind*III digested pJK11. The expression cassette was excised by digestion with *Spe*I and *Xho*I and inserted into the binary vector pPZPnat1 digested with *Spe*I and *Xho*I, creating the vector pPZPnat1-*LmHP1*. The pPZPnat1 vector, that contains the nourseothricin resistant gene *NAT1* (nourseothricin acetyltransferase gene), is used for gene silencing in *L. maculans via* agro-transformation. The same strategy was used to obtain the pPZPnat1-*LmDIM5* vector; the sense fragment was obtained using primers Silent*LmDIM5-Hind*III+ and Silent*LmDIM5-Bam*HI−; the antisense fragment using primers Silent*LmDIM5-Bam*HI+ and Silent*LmDIM5-Xma*I-.

Vector pBHt2-*LmHP1*-GFP was created by fusing the GFP coding sequence (p-EGFP-1, Clontech) downstream the coding sequence of *LmHP1* under the control of its own promoter in the binary vector pBHt2 containing the hygromycin B resistance gene (*hph*). The *LmHP1* entire sequence containing 5′ and 3′ UTR was amplified from cDNA of v23.1.3 mycelium using primers *LmHP1*-*Eco*RI and *LmHP1*-*Xba*I digested with *Eco*RI and *Xba*I and ligated into an *Eco*RI-*Xba*I digested pBHt2. The stop codon was excised by amplification of the pBHt2-*LmHP1* vector with C-*LmHP1*-*Spe*I and C-*LmHP1-Cla*I. The GFP coding sequence was amplified eGFP-*Cla*I and C-eGFP-*Spe*I from the p-EGFP-1 plasmid, digested with *Cla*I and *Spe*I and ligated into a *Cla*I-*Spe*I pBHt2-*LmHP1* digested vector, thus creating pBHt2-*LmHP1*-GFP vector. Primers used are detailed in Table S8.

The constructs were introduced into *A. tumefaciens* strain C58 by electroporation (1.5 kV, 200 ohms and 25 µF). *A. tumefaciens*-mediated transformation (ATMT) of *L. maculans* was performed as previously described [99] with minor modifications [19]. Transformants were plated on minimal media complemented with nourseothricin (50 mg/l) for pPZPnat1-*LmHP1* and pPZPnat1-*LmDIM5* or hygromycin (50 mg/l) for pBHt2-*LmHP1*-GFP and cefotaxime (250 mg/l).

### Confocal microscopy

Axenic mycelia from a Petri dish with minimal medium were stained by incubating for 15 min in 10 µg/µl DAPI solution (Sigma-Aldrich) at room temperature to observe nuclei. Analyses were performed on a Leica TCS SPE laser scanning confocal microscope. Excitation of GFP was at 488 nm and emission was captured with a 505–530 nm broad pass filter. DAPI was excited at 543 nm and emission was captured with a 580–615 nm broad pass filter. The detector gain was 800 with an amplifier offset from −0.2. All images represent the average of 4 scans.

### *In silico* analyses and gene annotations

HP1 and DIM-5 orthologs were identified with the NCBI BLAST program http://blast.ncbi.nlm.nih.gov/Blast.cgi [100]; functional domains have been identified using InterProScan http://www.ebi.ac.uk/Tools/pfa/iprscan. Annotation of untranslated regions (UTR), transcriptional start and stop sites and intron positions were performed following PCR amplification and sequencing of 3′- and 5′-ends of cDNA using Creator SMART cDNA Library Construction Kit (Clontech, Palo Alto, CA) according to manufacturer's recommendation and using HP1-5UTRL1, HP1-5UTRL2, HP1-3UTRU1, HP1-3UTRU2 and DIM5-5UTRL1, DIM5-5UTRL2, DIM5-3UTRU2; DIM5-3UTRU1 as specific primers (Table S8). Hierarchical clustering analyses were carried out using GENESIS [101], multiple alignements with COBALT [102].

### Statistical analyses

Disease scorings and radial growth of each isolate were analysed using ANOVA. For qRT-PCR analyses, Ct_target_ gene-Ct_βLmtubuline_ was calculated for each isolate and analysed using ANOVA. Isolates were compared using a Fisher test (α = 0.05). Transformants were compared to v23.1.3 by a Dunnet multiple comparison test (α = 0.05). All statistical analyses were performed using XLStat 7.5 software.

## Supporting Information

Figure S1 Dimethylation of lysine 4 of histone 3 (H3K4me2) in v23.1.3 and in silenced-*LmHP1* and silenced-*LmDIM5* background. Chromatin immunoprecipitation analysis was performed as control, using H3K4me2 mark in (A) the coding region of H2A, (B) the promoters of *AvrLm1* and *AvrLm4-7* or (C) the coding regions of *AvrLm1* and *AvrLm4-7*. Data were normalised using the “percent of input” method. AvrLm1_Pro and AvrLm4-7_Pro: promoter regions of *AvrLm1* and *AvrLm4-7*. Error bars indicate the standard deviation of two biological and two technical repeats.
(TIFF)

Table S1 List of *L. maculans* genes down-regulated in a silenced-*LmHP1* background.
(PDF)

Table S2 List of *L. maculans* genes down-regulated in a silenced-*LmDIM5* background.
(PDF)

Table S3 List of *L. maculans* genes up-regulated in a silenced-*LmDIM5* background.
(PDF)

Table S4 List of *L. maculans* genes up-regulated in a silenced-*LmHP1* background.
(PDF)

Table S5 List of genes up-regulated in both a silenced-*LmDIM5* and in a silenced-*LmHP1* background.
(PDF)

Table S6 Number of sequences generated and exploitable following TAIL-PCR experiments on transformants v29.3.1-*AvrLm1*, v29.3.1-*AvrLm6*, v29.3.1-*LmCys2* and NzT4-*AvrLm4-7*.
(PDF)

Table S7 Characteristics of the sequences obtained by TAIL-PCR.
(PDF)

Table S8 List of PCR primers used in this study.
(PDF)
