# Supplementary figures and images for: Epigenetic Control of Effector Gene Expression in the Plant Pathogenic Fungus Leptosphaeria maculans
Source: PLoS Genet. 2014 Mar 6;10(3):e1004227. doi: 10.1371/journal.pgen.1004227 (PMC3945186; doi:10.1371/journal.pgen.1004227)

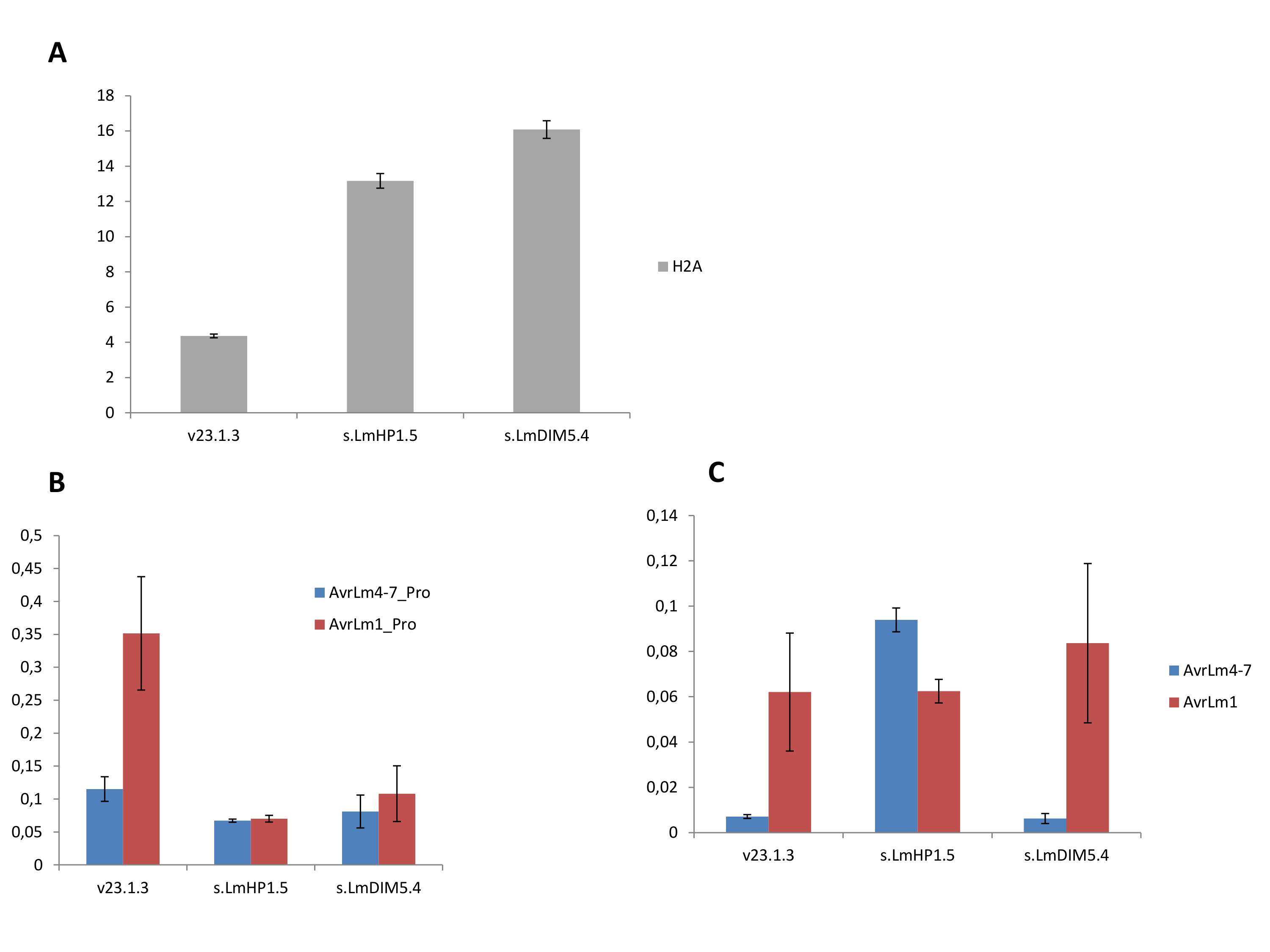

Supplement: Figure S1 — Dimethylation of lysine 4 of histone 3 (H3K4me2) in v23.1.3 and in silenced-LmHP1 and silenced-LmDIM5 background. Chromatin immunoprecipitation analysis was performed as control, using H3K4me2 mark in (A) the coding region of H2A, (B) the promoters of AvrLm1 and AvrLm4-7 or (C) the coding regions of AvrLm1 and AvrLm4-7. Data were normalised using the “percent of input” method. AvrLm1_Pro and AvrLm4-7_Pro: promoter regions of AvrLm1 and AvrLm4-7. Error bars indicate the standard deviation of two biological and two technical repeats. (TIFF) [file pgen.1004227.s001.tif]
