# Supplementary material for: Epigenetic Control of Effector Gene Expression in the Plant Pathogenic Fungus Leptosphaeria maculans
Source: PLoS Genet. 2014 Mar 6;10(3):e1004227. doi: 10.1371/journal.pgen.1004227 (PMC3945186; doi:10.1371/journal.pgen.1004227)
Supplement: Table S1 — List of L. maculans genes down-regulated in a silenced-LmHP1 background. (PDF) [file pgen.1004227.s002.pdf]

Table S1. List of *L. maculans* genes down-regulated in a silenced-*LmHP1* background.

SEQ_ID [a]	Fold change in silenced-*LmHP1*[b]	Location[c]	Function
lmctg_1317_v2_egn4_Lema_P094000.1	-62,2	AT-B	similar to dynamin
lmctg_1136_v2_egn4_Lema_P081070.1	-15,68	AT-B	putative SSP-encoding gene
lmctg_1476_v2_egn4_Lema_P107790.1	-11,42	GC-island	similar to acyl-protein thioesterase
lmctg_0056_v2_egn4_Lema_P005920.1	-11,07	GC-island	similar to glutathione-dependent formaldehyde-activating
lmctg_1613_v2_egn4_Lema_P121920.1	-10,31	AT-B	similar to glycosyl hydrolase
lmctg_0606_v2_egn4_Lema_P044950.1	-8,41	GC	similar to glucooligosaccharide oxidase
lmctg_0151_v2_egn4_Lema_P015290.1	-6,28	GC	putative SSP-encoding gene
lmctg_0689_v2_egn4_Lema_P049950.1	-5,65	GC-island	LmCys1
lmctg_1218_v2_egn4_Lema_P086310.1	-5,19	AT-B	putative SSP-encoding gene
lmctg_1149_v2_egn4_Lema_P081900.1	-4,72	GC	similar to UVI-1h
lmctg_0897_v2_egn4_Lema_P067200.1	-4,39	GC-island	similar to peptide synthase protein
lmctg_1213_v2_egn4_Lema_P086240.1	-4,37	AT-B	similar to putative peptidase
lmctg_0908_v2_egn4_Lema_P071390.1	-4,31	GC	similar to laccase
lmctg_0906_v2_egn4_Lema_P070500.1	-3,57	GC	similar to Glucan 1,3-beta-glucosidase precursor (Exo-1,3-beta-glucanase)
lmctg_0920_v2_egn4_Lema_P072930.1	-3,54	AT-B	similar to integral membrane protein
lmctg_1057_v2_egn4_Lema_P077510.1	-3,48	GC	similar to sporulation associated protein
lmctg_0078_v2_egn4_Lema_P008940.1	-3,39	AT-B	similar to ankyrin repeat protein
lmctg_0906_v2_egn4_Lema_P070590.1	-3,17	GC	similar to alpha/beta hydrolase fold family protein

lmctg_0066_v2_egn4_Lema_P006620.1	-3,13	GC	similar to EF-hand calcium-binding domain protein
lmctg_0023_v2_egn4_Lema_P002020.1	-2,81	GC	similar to 3-hydroxyisobutyrate dehydrogenase
lmctg_0377_v2_egn4_Lema_P026200.1	-2,73	GC	similar to telomeric DNA binding protein
lmctg_0948_v2_egn4_Lema_P073920.1	-2,57	GC	similar to protein kinase domain-containing protein
lmctg_1229_v2_egn2_Lema_uP085500.1	-2,55	AT-HB	putative SSP-encoding gene
lmctg_0872_v2_egn4_Lema_P062850.1	-2,52	GC	similar to nitrosoguanidine resistance protein
lmctg_1497_v2_egn4_Lema_P111550.1	-2,51	GC	similar to choline dehydrogenase
lmctg_1582_v2_egn4_Lema_P118810.1	-2,5	GC	similar to carbonic anhydrase
lmctg_1229_v2_egn4_Lema_uP086550.1	-2,41	AT-HB	putative SSP-encoding gene
lmctg_1335_v2_egn4_Lema_P098380.1	-2,39	GC	similar to major Facilitator superfamily protein
lmctg_0148_v2_egn4_Lema_P015080.1	-2,36	GC	putative SSP-encoding gene
lmctg_1058_v2_egn4_Lema_P077520.1	-2,32	GC	similar to fatty acid oxygenase
lmctg_1237_v2_egn4_Lema_P086820.1	-2,32	GC	putative SSP-encoding gene
lmctg_0347_v2_egn4_Lema_P024230.1	-2,27	GC	similar to MFS allantoate transporter
lmctg_0225_v2_egn4_Lema_P018230.1	-2,25	GC	similar to formate/nitrite transporter
lmctg_0948_v2_egn4_Lema_P073910.1	-2,19	GC	similar to DNA repair protein rad5
lmctg_0895_v2_egn4_Lema_P066700.1	-2,16	GC	similar to ATP-dependent clp protease atp-binding subunit clpx
lmctg_0550_v2_egn4_Lema_P035610.1	-2,15	GC	similar to putative peptide transporter
lmctg_0879_v2_egn4_Lema_P064550.1	-2,12	GC	similar to FAD-linked sulfhydryl oxidase ERV2
lmctg_0084_v2_egn4_Lema_P009270.1	-2,07	GC	putative SSP-encoding gene
lmctg_0904_v2_egn4_Lema_P070080.1	-2,02	GC	similar to isochorismatase hydrolase

lmctg_1212_v2_egn4_Lema_P085620.1	-2,02	AT-B	similar to cytochrome P450 monooxygenase
lmctg_1492_v2_egn4_Lema_P111480.1	-2,01	AT-B	similar to aldo/keto reductase
lmctg_0789_v2_egn4_Lema_uP059110.1	-2	GC	similar to GABA permease
lmctg_1476_v2_egn4_Lema_P108180.1	-1,98	GC	similar to MFS transporter
lmctg_0247_v2_egn4_Lema_P019560.1	-1,97	GC	similar to serine/threonine-protein kinase
lmctg_0788_v2_egn4_Lema_P059100.1	-1,96	GC	similar to GABA permease
lmctg_1463_v2_egn4_Lema_P105680.1	-1,95	GC	similar to beta-glucosidase
lmctg_0555_v2_egn4_Lema_P037040.1	-1,94	GC	similar to exoglucanase 1 precursor
lmctg_1149_v2_egn4_Lema_P081980.1	-1,94	GC	similar to salicylate hydroxylase
lmctg_1053_v2_egn4_Lema_P077260.1	-1,92	GC	similar to SWIM zinc finger family protein
lmctg_1098_v2_egn4_Lema_P079340.1	-1,88	GC	similar to hemerythrin HHE cation binding domain-containing protein
lmctg_1326_v2_egn4_Lema_P096290.1	-1,85	GC	similar to homologous pairing protein
lmctg_1237_v2_egn4_Lema_P086880.1	-1,84	GC	putative SSP-encoding gene
lmctg_1497_v2_egn4_Lema_P111510.1	-1,84	GC	similar to MFS transporter
lmctg_1660_v2_egn4_Lema_P123910.1	-1,82	GC-island	similar to putative transposase
lmctg_0541_v2_egn4_Lema_P033140.1	-1,8	GC	putative SSP-encoding gene
lmctg_0632_v2_egn4_Lema_P047300.1	-1,78	GC	similar to dihydroceramide delta-4 desaturase
lmctg_1621_v2_egn4_Lema_P123090.1	-1,77	GC	putative SSP-encoding gene
lmctg_1533_v2_egn4_Lema_P115450.1	-1,76	GC	similar to probable sterol C-24 reductase
lmctg_0050_v2_egn4_Lema_P004960.1	-1,75	GC	similar to CFEM domain-containing protein
lmctg_0176_v2_egn4_Lema_P015910.1	-1,75	GC	similar to pantothenate transporter
lmctg_0347_v2_egn4_Lema_P024220.1	-1,75	GC	similar to membrane transporter
lmctg_1244_v2_egn4_Lema_P089250.1	-1,75	GC	similar to malate dehydrogenase

lmctg_1294_v2_egn4_Lema_P092430.1	-1,74	GC	similar to TPA: Putative Zn(II)2Cys6 transcription factor (Eurofung)
lmctg_0033_v2_egn4_Lema_P003480.1	-1,73	GC	similar to structure-specific endonuclease catalytic subunit
lmctg_1475_v2_egn4_Lema_P107780.1	-1,73	GC-island	similar to phosphotransferase enzyme family protein
lmctg_1219_v2_egn4_Lema_P086330.1	-1,71	GC	similar to sodium bile acid symporter family protein
lmctg_0575_v2_egn4_Lema_P041050.1	-1,7	GC	similar to mitochondrial dicarboxylate carrier
lmctg_0021_v2_egn4_Lema_P001870.1	-1,69	GC	putative SSP-encoding gene
lmctg_0148_v2_egn4_Lema_P015100.1	-1,69	GC	putative SSP-encoding gene
lmctg_1570_v2_egn4_Lema_P118080.1	-1,69	GC	similar to histidine acid phosphatase
lmctg_0649_v2_egn4_Lema_P048640.1	-1,68	GC	similar to aconitate hydratase
lmctg_1613_v2_egn4_Lema_P121840.1	-1,68	GC	similar to cytochrome P450 monooxygenase
lmctg_1333_v2_egn4_Lema_P098080.1	-1,66	AT-B	similar to protein kinase
lmctg_0723_v2_egn4_Lema_uP054850.1	-1,63	GC	putative SSP-encoding gene
lmctg_0707_v2_egn4_Lema_P052270.1	-1,62	AT-B	similar to gryzun
lmctg_1438_v2_egn4_Lema_P102810.1	-1,62	GC	similar to MFS lactose permease
lmctg_1488_v2_egn4_Lema_P110830.1	-1,62	GC	similar to oxidoreductase
lmctg_0120_v2_egn4_Lema_uP013250.1	-1,61	GC	putative SSP-encoding gene
lmctg_1609_v2_egn4_Lema_P121030.1	-1,61	GC	similar to siderophore biosynthesis protein
lmctg_1489_v2_egn4_Lema_P111140.1	-1,6	GC	similar to importin beta-4 subunit
lmctg_1322_v2_egn4_Lema_P094950.1	-1,59	GC	similar to kelch repeats protein
lmctg_0052_v2_egn4_Lema_P005290.1	-1,58	GC	similar to acetyltransferase
lmctg_0091_v2_egn4_Lema_P010580.1	-1,58	GC	similar to phosphoglucomutase
lmctg_1031_v2_egn4_Lema_P076480.1	-1,58	GC	similar to amino acid transporter
lmctg_0064_v2_egn4_Lema_P006490.1	-1,57	GC	similar to HEAT repeat protein

			(DRIM)
lmctg_0103_v2_egn4_Lema_P012260.1	-1,56	GC	similar to osmosensor protein
lmctg_0906_v2_egn4_Lema_P071080.1	-1,56	GC	similar to purine utilization regulatory protein
lmctg_1536_v2_egn4_Lema_P115920.1	-1,56	GC	similar to carboxylesterase
lmctg_0592_v2_egn4_Lema_P043290.1	-1,55	GC	similar to dynamin
lmctg_0718_v2_egn4_Lema_P054260.1	-1,55	GC	similar to phosphatidylinositol phospholipase C
lmctg_1249_v2_egn4_Lema_P090090.1	-1,55	GC	similar to cytoplasm protein
AT02_ext_SuperContig_22_10	-1,54	AT-B	putative SSP-encoding gene
lmctg_0103_v2_egn4_Lema_P012150.1	-1,54	GC	similar to longevity-assurance protein
lmctg_0332_v2_egn4_Lema_P023620.1	-1,54	GC	similar to protein kinase
lmctg_0407_v2_egn4_Lema_P028320.1	-1,53	AT-B	similar to dtdp-glucose 4,6-dehydratase
lmctg_1369_v2_egn4_Lema_P099800.1	-1,52	GC	similar to serine-rich protein
lmctg_1533_v2_egn4_Lema_P115350.1	-1,52	GC	similar to K(+)/H(+) antiporter
lmctg_1570_v2_egn4_Lema_P118000.1	-1,52	GC	similar to phosphotransferase enzyme family protein
lmctg_1354_v2_egn4_Lema_P099590.1	-1,51	GC	similar to serine/threonine protein kinase
lmctg_1605_v2_egn4_Lema_P120500.1	-1,51	GC	putative SSP-encoding gene
lmctg_1525_v2_egn4_Lema_P114650.1	-1,5	GC	similar to isoleucyl-tRNA synthetase

[a] Only genes encoding proteins with a predicted function are presented.

[b] Genes with fold change <-1.5 in transcript level and an associated *p* value<0.05 were considered as significantly up-regulated the silenced-*LmDIM5* transformant compared to the wild type v23.1.3 isolate in axenic culture.

[c] GC refers to GC-isochores; AT-HB refers to AT-isochores; AT-B refers to 859(±385) bp transition regions between AT-isochores and GC-isochores; GC-islands refer to regions of more than 1 kb within AT-isochores with a GC content > 50%.
