# Supplementary material for: Epigenetic Control of Effector Gene Expression in the Plant Pathogenic Fungus Leptosphaeria maculans
Source: PLoS Genet. 2014 Mar 6;10(3):e1004227. doi: 10.1371/journal.pgen.1004227 (PMC3945186; doi:10.1371/journal.pgen.1004227)
Supplement: Table S2 — List of L. maculans genes down-regulated in a silenced-LmDIM5 background. (PDF) [file pgen.1004227.s003.pdf]

Table S2. List of *L. maculans* genes down-regulated in a silenced-*LmDIM5* background

SEQ_ID [a]	Fold Change in silenced-*LmDIM5* [b]	Location [c]	Function
lmctg_1317_v2_egn4_Lema_P094000.1	-214,14	AT-B	similar to dynamin
AT01_ext_SuperContig_29_7	-36,69	AT-B	putative SSP-encoding gene
lmctg_1136_v2_egn4_Lema_P081070.1	-35,26	AT-B	putative SSP-encoding gene
lmctg_0056_v2_egn4_Lema_P005920.1	-26,28	GC-island	similar to glutathione-dependent formaldehyde-activating
lmctg_0563_v2_egn4_Lema_P038280.1	-20	AT-B	similar to putative pathogenesis related protein
lmctg_1136_v2_egn4_Lema_P081170.1	-18,56	GC	similar to MFS monosaccharide transporter
lmctg_1187_v2_egn4_Lema_P083940.1	-15,42	GC	putative SSP-encoding gene
lmctg_1156_v2_egn4_Lema_P082180.1	-12,41	GC	similar to salicylate hydroxylase
lmctg_1157_v2_egn4_Lema_P082270.1	-11,94	GC	similar to trypsin
lmctg_1476_v2_egn4_Lema_P107790.1	-11,55	GC-island	similar to acyl-protein thioesterase
lmctg_1136_v2_egn4_Lema_P081180.1	-10,54	GC	putative SSP-encoding gene
lmctg_0252_v2_egn4_Lema_P019640.1	-9,9	GC	similar to carbonic anhydrase
lmctg_0276_v2_egn4_Lema_P021120.1	-9,89	GC	putative SSP-encoding gene
lmctg_1317_v2_egn4_Lema_P093980.1	-9,77	GC	similar to beta-galactosidase
lmctg_0889_v2_egn4_Lema_P065710.1	-9,72	GC	similar to Arylsulfotransferase (ASST)
lmctg_0048_v2_egn4_Lema_P004740.1	-9,71	AT-B	similar to phosphate-repressible phosphate permease
lmctg_0689_v2_egn4_Lema_P049950.1	-9,62	GC-island	LmCys1
lmctg_0021_v2_egn4_Lema_P001870.1	-9,59	GC	putative SSP-encoding gene
lmctg_0606_v2_egn4_Lema_P044950.1	-9,02	GC	similar to glucooligosaccharide oxidase
lmctg_1487_v2_egn4_Lema_P110770.1	-8,22	AT-B	similar to transporter
lmctg_1532_v2_egn4_Lema_P115190.1	-7,93	GC	similar to sugar transporter
lmctg_0146_v2_egn4_Lema_P014740.1	-7,73	GC	similar to ABC-transporter
lmctg_1149_v2_egn4_Lema_P081980.1	-7,67	GC	similar to salicylate hydroxylase

lmctg_0924_v2_egn4_Lema_P073010.1	-7,31	GC	similar to salicylate hydroxylase
lmctg_0908_v2_egn4_Lema_P071330.1	-7,04	GC	similar to MFS transporter
lmctg_1613_v2_egn4_Lema_P121920.1	-6,21	AT-B	similar to glycosyl hydrolase
lmctg_0606_v2_egn4_Lema_P044940.1	-5,78	GC	similar to MFS transporter
lmctg_1218_v2_egn4_Lema_P086310.1	-5,53	AT-B	putative SSP-encoding gene
lmctg_1317_v2_egn4_Lema_P093950.1	-5,11	GC	similar to amidohydrolase
lmctg_0078_v2_egn4_Lema_P008940.1	-5,01	AT-B	similar to ankyrin repeat protein
lmctg_0920_v2_egn4_Lema_P072930.1	-4,93	AT-B	similar to integral membrane protein
lmctg_0719_v2_egn4_Lema_P054450.1	-4,91	AT-B	similar to homogentisate 1,2-dioxygenase
lmctg_0119_v2_egn4_Lema_P013210.1	-4,9	GC	putative SSP-encoding gene
lmctg_1475_v2_egn4_Lema_P107780.1	-4,84	GC-island	similar to phosphotransferase enzyme family protein
lmctg_1136_v2_egn4_Lema_P081000.1	-4,81	AT-B	similar to cytochrome P450
lmctg_1326_v2_egn4_Lema_P097030.1	-4,76	GC	putative SSP-encoding gene
lmctg_0707_v2_egn4_Lema_P052110.1	-4,45	GC	similar to cytochrome P450
lmctg_0872_v2_egn4_Lema_P062850.1	-4,26	GC	similar to nitrosoguanidine resistance protein
lmctg_0906_v2_egn4_Lema_P070590.1	-4,22	GC	similar to alpha/beta hydrolase fold family protein
lmctg_1531_v2_egn4_Lema_P114820.1	-4,07	AT-B	similar to ascorbate oxidase
lmctg_1317_v2_egn4_Lema_P093990.1	-4,06	GC	similar to YesU
lmctg_0762_v2_egn4_Lema_P058320.1	-4,05	GC	similar to trehalase
lmctg_1497_v2_egn4_Lema_P111550.1	-4,02	GC	similar to choline dehydrogenase
lmctg_1492_v2_egn4_Lema_P111480.1	-3,98	AT-B	similar to aldo/keto reductase
lmctg_0333_v2_egn4_Lema_P023670.1	-3,84	GC	similar to folylpolyglutamate synthase
lmctg_1037_v2_egn4_Lema_P076850.1	-3,64	GC	similar to tyrosinase
lmctg_0095_v2_egn4_Lema_P011350.1	-3,61	GC	similar to catechol dioxygenase
lmctg_0044_v2_egn4_Lema_P004720.1	-3,58	AT-HB	putative SSP-encoding gene
lmctg_0868_v2_egn4_Lema_P062480.1	-3,54	AT-B	similar to integral membrane protein
lmctg_0574_v2_egn4_Lema_P040920.1	-3,51	GC	similar to hexose transporter
lmctg_0053_v2_egn4_Lema_P005640.1	-3,5	GC	putative SSP-encoding gene
AT01_ext_SuperContig_9_1	-3,46	AT-B	putative SSP-encoding gene
lmctg_1335_v2_egn4_Lema_P098380.1	-3,44	GC	similar to major Facilitator superfamily protein

lmctg_0103_v2_egn4_Lema_P012400.1	-3,13	GC	similar to siderophore iron transporter
lmctg_1565_v2_egn4_Lema_P117520.1	-3,12	GC	similar to benomyl/methotrexate resistance protein
lmctg_0056_v2_egn4_Lema_P005960.1	-2,99	AT-B	similar to glutathione S-transferase
lmctg_1459_v2_egn4_Lema_P104520.1	-2,98	AT-B	similar to GABA permease
lmctg_1570_v2_egn4_Lema_P117990.1	-2,87	GC	similar to putative efflux pump
lmctg_1212_v2_egn4_Lema_P085620.1	-2,87	AT-B	similar to cytochrome P450 monooxygenase
lmctg_0906_v2_egn4_Lema_uP070610.1	-2,79	GC	putative SSP-encoding gene
lmctg_0616_v2_egn4_Lema_uP045630.1	-2,77	GC	putative SSP-encoding gene
lmctg_0736_v2_egn4_Lema_P056310.1	-2,76	GC	similar to lactonohydrolase
lmctg_1307_v2_egn4_Lema_P093650.1	-2,76	GC	similar to FAD binding domain protein
lmctg_0433_v2_egn4_Lema_P028930.1	-2,71	GC	similar to threonine aldolase
lmctg_1431_v2_egn4_Lema_P102180.1	-2,71	GC	similar to ornithine aminotransferase
lmctg_1523_v2_egn4_Lema_P113240.1	-2,71	AT-B	similar to onanonoxo-7-onima-8-eninoihtemlysoneda
lmctg_1326_v2_egn4_Lema_P096950.1	-2,62	GC	similar to Taurine catabolism dioxygenase TauD
lmctg_0599_v2_egn4_Lema_P044030.1	-2,61	GC	similar to proteinase (secreted protein)
lmctg_1246_v2_egn4_Lema_P089400.1	-2,53	GC	similar to GMC oxidoreductase
lmctg_1250_v2_egn4_Lema_P090190.1	-2,53	GC	similar to MFS multidrug transporter
lmctg_0714_v2_egn4_Lema_P053110.1	-2,52	GC	similar to phosphomevalonate kinase
lmctg_1667_v2_egn4_Lema_P124320.1	-2,52	GC	putative SSP-encoding gene
lmctg_1220_v2_egn4_Lema_P086420.1	-2,51	GC	similar to sugar transporter
lmctg_0555_v2_egn4_Lema_P037040.1	-2,5	GC	similar to exoglucanase 1 precursor
lmctg_1322_v2_egn4_Lema_P094950.1	-2,5	GC	similar to kelch repeats protein
lmctg_0050_v2_egn4_Lema_P004960.1	-2,47	GC	similar to CFEM domain-containing protein
lmctg_0453_v2_egn4_Lema_P030350.1	-2,43	GC	similar to pigment biosynthesis protein Ayg1
lmctg_0469_v2_egn4_Lema_P031000.1	-2,43	GC	similar to NAD-specific glutamate dehydrogenase
lmctg_0560_v2_egn4_Lema_P037670.1	-2,41	GC	similar to FAD dependent oxidoreductase
lmctg_0078_v2_egn4_Lema_P008930.1	-2,4	AT-B	similar to NACHT and Ankyrin domain protein
lmctg_0477_v2_egn4_Lema_P031600.1	-2,39	GC	similar to ThiJ/PfpI
lmctg_0915_v2_egn4_Lema_P072630.1	-2,39	GC	similar to transcriptional activator xlnR
lmctg_0076_v2_egn4_Lema_P008080.1	-2,35	GC	similar to MFS transporter

lmctg_1233_v2_egn4_Lema_P086730.1	-2,28	GC	similar to polyketide synthase
lmctg_0718_v2_egn4_Lema_P053880.1	-2,26	GC	similar to clc channel
lmctg_0876_v2_egn4_Lema_P063120.1	-2,24	GC	similar to secretory component protein shr3
lmctg_1613_v2_egn4_Lema_P121840.1	-2,24	GC	similar to cytochrome P450 monooxygenase
lmctg_0801_v2_egn4_Lema_P059620.1	-2,21	GC	similar to FAD binding domain protein
lmctg_0902_v2_egn4_Lema_P069300.1	-2,21	GC	similar to acetamidase
lmctg_0015_v2_egn4_Lema_P000920.1	-2,2	GC	similar to neutral ceramidase
lmctg_1265_v2_egn4_Lema_P091740.1	-2,2	GC	similar to oxidoreductase
lmctg_1276_v2_egn4_Lema_P092110.1	-2,2	GC	similar to iron-regulated transporter
lmctg_0048_v2_egn4_Lema_P004750.1	-2,19	AT-B	similar to phosphoribosylformylglycinamidine synthase
lmctg_0707_v2_egn4_Lema_P052170.1	-2,18	GC	similar to siderophore iron transporter
lmctg_0717_v2_egn4_Lema_P053640.1	-2,16	GC	putative SSP-encoding gene
lmctg_0332_v2_egn4_Lema_P023620.1	-2,14	GC	similar to protein kinase
lmctg_0919_v2_egn4_Lema_P072900.1	-2,14	GC	similar to nonribosomal peptide synthase
lmctg_1500_v2_egn4_Lema_P112200.1	-2,13	GC	similar to tRNA methyltransferase
lmctg_1533_v2_egn4_Lema_P115350.1	-2,13	GC	similar to K(+)/H(+) antiporter
lmctg_0707_v2_egn4_Lema_P052270.1	-2,12	AT-B	similar to gryzun
lmctg_0372_v2_egn4_Lema_P025790.1	-2,11	GC	similar to cystathionine-gamma-lyase
lmctg_1609_v2_egn4_Lema_P120950.1	-2,11	AT-B	similar to bleomycin hydrolase
lmctg_0066_v2_egn4_Lema_P006900.1	-2,1	GC	similar to endo-beta-1,4-mannanase
lmctg_0260_v2_egn4_Lema_P020150.1	-2,1	GC	similar to carboxypeptidase S1
lmctg_1478_v2_egn4_Lema_P108950.1	-2,1	GC	similar to methyltransferase
lmctg_1524_v2_egn4_Lema_P114320.1	-2,1	GC	similar to Histidyl-tRNA synthetase
lmctg_1233_v2_egn4_Lema_P086740.1	-2,1	AT-B	similar to polyketide synthase
lmctg_0347_v2_egn4_Lema_P024220.1	-2,07	GC	similar to membrane transporter
lmctg_0545_v2_egn4_Lema_P035250.1	-2,07	GC	similar to ubiquitin C-terminal hydrolase L3
lmctg_0901_v2_egn4_Lema_P068460.1	-2,07	GC	similar to beta-glucosidase
lmctg_1437_v2_egn4_Lema_P102680.1	-2,07	GC	similar to amidohydrolase
lmctg_1477_v2_egn4_Lema_P108230.1	-2,07	GC	similar to mKIAA0829 protein
lmctg_0066_v2_egn4_Lema_P006650.1	-2,06	GC	similar to ATP dependent RNA helicase

lmctg_1143_v2_egn4_Lema_P081570.1	-2,04	GC	similar to NRPS-like enzyme
lmctg_1244_v2_egn4_Lema_P088810.1	-2,04	GC	similar to importin beta-2
lmctg_1476_v2_egn4_Lema_P108150.1	-2,04	GC	similar to fungal cellulose binding domain protein
lmctg_1532_v2_egn4_Lema_P115000.1	-2,04	GC	similar to peptidase S41 family protein
lmctg_0062_v2_egn4_Lema_P006140.1	-2,03	GC	similar to arginase
lmctg_0897_v2_egn4_Lema_P067100.1	-2,01	GC	similar to 4-aminobutyrate aminotransferase
lmctg_0904_v2_egn4_Lema_P070270.1	-2,01	GC	similar to PAB-dependent poly(A)-specific ribonuclease subunit (Pan2)
lmctg_1437_v2_egn4_Lema_P102700.1	-2,01	GC	similar to MFS transporter
lmctg_0750_v2_egn4_Lema_P057570.1	-2	GC	similar to methyltransferase
lmctg_0444_v2_egn4_Lema_P030050.1	-1,99	GC	similar to nitrilase
lmctg_1570_v2_egn4_Lema_P118080.1	-1,99	GC	similar to histidine acid phosphatase
lmctg_0120_v2_egn4_Lema_P013260.1	-1,97	GC	similar to endoglucanase
lmctg_0693_v2_egn4_Lema_P050570.1	-1,96	AT-B	similar to aldose reductase
lmctg_0151_v2_egn4_Lema_P015270.1	-1,95	GC	similar to choline dehydrogenase
lmctg_0279_v2_egn4_Lema_P021320.1	-1,95	GC	similar to beta-lactamase
lmctg_0824_v2_egn4_Lema_P061020.1	-1,94	GC	similar to L-galactose dehydrogenase (L-GalDH)
lmctg_1317_v2_egn4_Lema_P094090.1	-1,94	GC	similar to actin
lmctg_0628_v2_egn4_Lema_P047120.1	-1,93	GC	similar to guanylate kinase
lmctg_1249_v2_egn4_Lema_P090090.1	-1,93	GC	similar to cytoplasm protein
lmctg_0432_v2_egn4_Lema_P028880.1	-1,92	GC	similar to CFEM domain-containing protein
lmctg_1324_v2_egn4_Lema_P095380.1	-1,92	GC	similar to mitochondrial protein
lmctg_0023_v2_egn4_Lema_P001990.1	-1,9	GC	similar to alcohol dehydrogenase
lmctg_0103_v2_egn4_Lema_P012100.1	-1,9	GC	similar to hexose transporter
lmctg_1249_v2_egn4_Lema_P089930.1	-1,89	GC	similar to gi|259016461|sp|O13938,3|YEP4_SCHPO RecName: Full=UPF0658 Golgi apparatus membrane protein C23H3,04
AT03_ext_SuperContig_5_12	-1,89	GC-island	putative SSP-encoding gene
lmctg_0755_v2_egn4_Lema_P057990.1	-1,87	GC	similar to cystathionine beta-synthase
lmctg_0094_v2_egn4_Lema_P011220.1	-1,84	GC	similar to chromosome segregation protein sudA

lmctg_0095_v2_egn4_Lema_P011390.1	-1,84	GC	similar to phenylacetyl-CoA ligase
lmctg_0114_v2_egn4_Lema_P012970.1	-1,84	GC	similar to a-pheromone processing metallopeptidase Ste23
lmctg_0656_v2_egn4_Lema_P049130.1	-1,84	GC	putative SSP-encoding gene
lmctg_1467_v2_egn4_Lema_P106610.1	-1,84	GC-island	similar to tetracycline-efflux transporter
lmctg_0023_v2_egn4_Lema_P002000.1	-1,83	GC	similar to regulatory protein
lmctg_1036_v2_egn4_Lema_P076830.1	-1,82	GC	similar to DNA repair protein Rhp26/Rad26
lmctg_1486_v2_egn4_Lema_P110620.1	-1,82	GC	similar to ferric-chelate reductase
lmctg_0662_v2_egn4_Lema_P049360.1	-1,81	GC	similar to feruloyl esterase
lmctg_0717_v2_egn4_Lema_P053590.1	-1,81	GC	similar to DNA replication complex GINS protein psf1
lmctg_1219_v2_egn4_Lema_P086330.1	-1,81	GC	similar to sodium bile acid symporter family protein
lmctg_0918_v2_egn4_Lema_P072890.1	-1,81	AT-B	similar to nonribosomal peptide synthase
lmctg_1476_v2_egn4_Lema_P107910.1	-1,8	GC	similar to aldehyde dehydrogenase
lmctg_0550_v2_egn4_Lema_P035920.1	-1,78	GC	similar to glucoamylase
lmctg_1239_v2_egn4_Lema_P087060.1	-1,78	GC	similar to pseudouridine synthase
lmctg_0276_v2_egn4_Lema_P021100.1	-1,77	GC	similar to vacuolar protein sorting protein DigA
lmctg_0401_v2_egn4_Lema_P027600.1	-1,77	GC	similar to dynactin 2 (p50)
lmctg_0476_v2_egn4_Lema_P031400.1	-1,76	GC	similar to vacuolar transporter chaperone
lmctg_0524_v2_egn4_Lema_P033030.1	-1,76	GC	similar to Fungal specific transcription factor
lmctg_1333_v2_egn4_Lema_uP098130.1	-1,76	GC	putative SSP-encoding gene
lmctg_0612_v2_egn4_Lema_P045360.1	-1,75	GC	similar to UBX domain protein
lmctg_1261_v2_egn4_Lema_P091390.1	-1,75	GC	similar to DNA repair protein rad50
lmctg_1531_v2_egn4_Lema_P114860.1	-1,75	GC	similar to Glycylpeptide N-tetradecanoyltransferase
AT20_ext_SuperContig_0_5	-1,75	AT-B	putative SSP-encoding gene
lmctg_0789_v2_egn4_Lema_uP059110.1	-1,74	GC	similar to GABA permease
lmctg_0557_v2_egn4_Lema_P037200.1	-1,74	AT-B	similar to TPR domain-containing protein
lmctg_1476_v2_egn4_Lema_P107800.1	-1,74	AT-B	similar to vacuolar sorting protein
lmctg_1347_v2_egn4_Lema_P099230.1	-1,73	GC	similar to bifunctional purine biosynthesis protein
lmctg_1117_v2_egn4_Lema_P080520.1	-1,72	GC	similar to DNA helicase
lmctg_1532_v2_egn4_Lema_P115170.1	-1,72	AT-B	similar to cyclin-dependent protein kinase

lmctg_0584_v2_egn4_Lema_P042350.1	-1,71	GC	similar to kinetochore protein fta7
lmctg_1489_v2_egn4_Lema_P111230.1	-1,71	GC	similar to monooxygenase
lmctg_1564_v2_egn4_Lema_P117180.1	-1,71	GC	similar to zinc transporter protein
lmctg_1106_v2_egn4_Lema_P079800.1	-1,7	GC	similar to FacB protein
lmctg_0879_v2_egn4_Lema_P064550.1	-1,69	GC	similar to FAD-linked sulfhydryl oxidase ERV2
lmctg_0064_v2_egn4_Lema_P006390.1	-1,68	GC	similar to monooxygenase
lmctg_0889_v2_egn4_Lema_P065630.1	-1,68	GC	similar to eukaryotic translation initiation factor 3 subunit EifCj
lmctg_0349_v2_egn4_Lema_uP024630.1	-1,68	GC	similar to sn-1,2-diacylglycerol cholinephosphotransferase
lmctg_0456_v2_egn4_Lema_P030410.1	-1,68	AT-B	similar to DNA damage repair protein Mus42
lmctg_0034_v2_egn4_Lema_P004090.1	-1,67	GC	similar to glutamyl-tRNA synthetase
lmctg_0405_v2_egn4_Lema_P028200.1	-1,67	GC	similar to peroxisomal targeting signal receptor
lmctg_0915_v2_egn4_Lema_P072710.1	-1,67	GC	similar to ankyrin repeat protein
lmctg_1609_v2_egn4_Lema_P121030.1	-1,67	GC	similar to siderophore biosynthesis protein
lmctg_0242_v2_egn4_Lema_P019300.1	-1,66	GC	similar to alpha 1,6 mannosyltransferase
lmctg_0628_v2_egn4_Lema_P047170.1	-1,66	GC	similar to TPA: DUF431 domain protein (AFU_orthologue; AFUA_1G08950)
lmctg_0718_v2_egn4_Lema_P054260.1	-1,66	GC	similar to phosphatidylinositol phospholipase C
lmctg_0693_v2_egn4_Lema_P050390.1	-1,65	GC	similar to M protein repeat protein
lmctg_0718_v2_egn4_Lema_P054290.1	-1,65	GC	similar to acetylesterase
lmctg_1199_v2_egn4_Lema_P084570.1	-1,65	GC	similar to glutaminyl-tRNA synthetase
lmctg_0030_v2_egn4_Lema_P003040.1	-1,64	GC	similar to sodium P-type ATPase
lmctg_0950_v2_egn4_Lema_P074030.1	-1,64	GC	similar to riboflavin synthase
lmctg_1580_v2_egn4_Lema_P118580.1	-1,64	GC	similar to WD repeat protein
lmctg_0443_v2_egn4_Lema_P030010.1	-1,63	GC	similar to 5-oxoprolinase
lmctg_1253_v2_egn4_Lema_P090440.1	-1,63	GC	similar to lipase
lmctg_1467_v2_egn4_Lema_P106930.1	-1,63	AT-B	putative SSP-encoding gene
lmctg_0100_v2_egn4_Lema_P011870.1	-1,62	GC	similar to autophagy ubiquitin-activating enzyme ApgG
lmctg_0206_v2_egn4_Lema_P017380.1	-1,62	GC	similar to exosome complex exonuclease Rrp4

lmctg_0718_v2_egn4_Lema_P053820.1	-1,62	GC	similar to translation initiation factor RLI1
lmctg_1323_v2_egn4_Lema_P095050.1	-1,62	GC	similar to calcium/calmodulin-dependent protein kinase
lmctg_0910_v2_egn4_Lema_P072090.1	-1,62	AT-B	similar to S-adenosylmethionine-dependent methyltransferase
lmctg_0750_v2_egn4_Lema_P057620.1	-1,61	GC	similar to ABC bile acid transporter
lmctg_0941_v2_egn4_Lema_P073680.1	-1,6	GC	similar to phospho-2-dehydro-3-deoxyheptonate aldolase
lmctg_0749_v2_egn4_Lema_P057500.1	-1,59	GC	similar to methylenetetrahydrofolate reductase
lmctg_0788_v2_egn4_Lema_P059100.1	-1,59	GC	similar to GABA permease
lmctg_0889_v2_egn4_Lema_P066150.1	-1,59	GC	similar to Fungal Zn binuclear cluster domain containing protein
lmctg_0906_v2_egn4_Lema_P070820.1	-1,59	GC	similar to kinesin heavy chain
lmctg_1317_v2_egn4_Lema_P093910.1	-1,59	GC	similar to dihydrodipicolinate synthetase family protein
lmctg_0296_v2_egn4_Lema_uP022430.1	-1,59	GC	similar to HLA class III protein Dom3z
lmctg_0429_v2_egn4_Lema_P028710.1	-1,58	GC	putative SSP-encoding gene
lmctg_0870_v2_egn4_Lema_P062710.1	-1,58	GC	similar to ARF GTPase activator (Csx2)
lmctg_1609_v2_egn4_Lema_P121010.1	-1,57	GC	similar to adenylosuccinate lyase
lmctg_0353_v2_egn4_Lema_P025230.1	-1,56	GC	similar to benzodiazepine receptor family protein
lmctg_1253_v2_egn4_Lema_P090580.1	-1,56	GC	similar to histidine biosynthesis protein
lmctg_0253_v2_egn4_Lema_P019720.1	-1,55	GC	similar to folate carrier protein
lmctg_0295_v2_egn4_Lema_P022420.1	-1,55	GC	similar to protein rai1
lmctg_0563_v2_egn4_Lema_P038630.1	-1,55	GC	similar to steroid alpha reductase
lmctg_0635_v2_egn4_Lema_P047740.1	-1,55	GC	similar to elongator complex protein
lmctg_1294_v2_egn4_Lema_P092430.1	-1,55	GC	similar to TPA: Putative Zn(II)2Cys6 transcription factor (Eurofung)
lmctg_0275_v2_egn4_Lema_P021090.1	-1,54	GC	similar to vacuolar protein sorting protein DigA
lmctg_0557_v2_egn4_Lema_P037220.1	-1,54	GC	similar to enoyl-CoA hydratase/isomerase
lmctg_1409_v2_egn4_Lema_P100730.1	-1,54	GC	similar to tubulin-folding cofactor B

lmctg_1570_v2_egn4_Lema_P117890.1	-1,54	GC	similar to gi|254763264|sp|Q5B3U7,2|CSN2_EMENI RecName: Full=COP9 signalosome complex subunit 2; Short=Signalosome subunit 2
lmctg_0554_v2_egn4_Lema_P036930.1	-1,53	GC	similar to rho-gdp dissociation inhibitor
lmctg_1003_v2_egn4_Lema_P076150.1	-1,53	GC	similar to fructosyl-amino acid oxidase
lmctg_1331_v2_egn4_Lema_P097780.1	-1,53	GC	similar to class I alpha-mannosidase
lmctg_1438_v2_egn4_Lema_P102800.1	-1,53	GC	similar to beta-galactosidase
lmctg_1533_v2_egn4_Lema_P115530.1	-1,53	GC	similar to integral membrane protein
lmctg_0608_v2_egn4_Lema_P045140.1	-1,52	GC	similar to glutathione reductase
lmctg_1531_v2_egn4_Lema_P114910.1	-1,52	GC	similar to ABC transporter
lmctg_0119_v2_egn4_Lema_P013200.1	-1,51	GC	similar to farnesyl-pyrophosphate synthetase
lmctg_0613_v2_egn4_Lema_P045380.1	-1,51	GC	similar to protein binding protein
lmctg_0904_v2_egn4_Lema_P070260.1	-1,51	GC	similar to nucleic acid binding
lmctg_0068_v2_egn4_Lema_P007180.1	-1,5	GC	similar to mitogen activated protein kinase 1
lmctg_0701_v2_egn4_Lema_P051200.1	-1,5	GC	similar to casein kinase II subunit beta
lmctg_0899_v2_egn4_Lema_P067710.1	-1,5	GC	similar to parallel beta-helix repeat protein
lmctg_1459_v2_egn4_Lema_P104570.1	-1,5	GC	similar to dihydroxy-acid dehydratase

[a] Only genes encoding proteins with a predicted function are presented.

[b] Genes with fold change <-1.5 in transcript level and an associated *p* value<0.05 were considered as significantly up-regulated the silenced-*LmDIM5* transformant compared to the wild type v23.1.3 isolate in axenic culture.

[c] GC refers to GC-isochores; AT-HB refers to AT-isochores; AT-B refers to 859(±385) bp transition regions between AT-isochores and GC-isochores; GC-islands refer to regions of more than 1 kb within AT-isochores with a GC content > 50%.
