# Supplementary material for: Epigenetic Control of Effector Gene Expression in the Plant Pathogenic Fungus Leptosphaeria maculans
Source: PLoS Genet. 2014 Mar 6;10(3):e1004227. doi: 10.1371/journal.pgen.1004227 (PMC3945186; doi:10.1371/journal.pgen.1004227)
Supplement: Table S3 — List of L. maculans genes up-regulated in a silenced-LmDIM5 background. (PDF) [file pgen.1004227.s004.pdf]

Table S3. List of *L. maculans* genes up-regulated in a silenced-*LmDIM5* background

SEQ_ID [a]	Fold change [b]	Location [c]	Function
lmctg_0034_v2_egn4_Lema_uP004230.1	104.40	GC	putative SSP-encoding gene
lmctg_0001_v2_T000001	77.16	AT-HB	putative SSP-encoding gene
lmctg_0898_v2_T000001	62.19	AT-HB	putative SSP-encoding gene
lmctg_1228_v2_T000001	55.76	AT-B	putative SSP-encoding gene
lmctg_0105_v2_egn2_Lema_P012060.1	30.18	AT-HB	putative SSP-encoding gene
AT10_ext_SuperContig_10_G00134	17.45	AT-HB	putative SSP-encoding gene
AvrLm1	16.93	AT-HB	AvrLm1
lmctg_1214_v2_egn4_Lema_P086290.1	16.83	AT-HB	AvrLm4-7
lmctg_0034_v2_egn4_Lema_uP004240.1	14.64	GC	putative SSP-encoding gene
lmctg_1031_v2_egn4_Lema_P076380.1	14.56	AT-B	putative SSP-encoding gene
AT15_ext_SuperContig_5_3	11.45	AT-HB	putative SSP-encoding gene
lmctg_0448_v2_egn4_Lema_uP030130.1	8.91	GC	putative SSP-encoding gene
AT01_ext_SuperContig_6_G00099	8.64	AT-HB	putative SSP-encoding gene
lmctg_1349_v2_egn4_Lema_P099300.1	8.42	GC	putative SSP-encoding gene
lmctg_1451_v2_egn4_Lema_P103310.1	8.14	GC	candidate b-1,4-xylanase
lmctg_1229_v2_egn4_Lema_P086540.1	8.04	AT-HB	similar to Six5
lmctg_0905_v2_egn4_Lema_P070370.1	7.85	GC	putative SSP-encoding gene
lmctg_0372_v2_egn4_Lema_P025730.1	7.62	GC	similar to cytochrome P450
lmctg_0898_v2_egn4_Lema_uP067380.1	6.94	GC	putative SSP-encoding gene
lmctg_0034_v2_egn4_Lema_P003720.1	6.16	GC	similar to amidase
lmctg_0122_v2_egn4_Lema_P013520.1	5.96	GC	candidate b-fructosidase
lmctg_0610_v2_egn4_Lema_P045240.1	5.93	GC	similar to oxidase
lmctg_0810_v2_egn4_Lema_P060230.1	5.84	GC	similar to monosaccharide transporter
lmctg_0621_v2_egn4_Lema_P046400.1	5.81	GC	candidate endo-b-1,4-glucanase
lmctg_0544_v2_egn4_Lema_P035000.1	5.60	GC	similar to Six5
lmctg_1624_v2_egn4_Lema_P123290.1	5.55	GC	candidate chitin-binding protein; five CBM18 modules
lmctg_0810_v2_egn4_Lema_P060220.1	5.48	GC	similar to sugar transporter
lmctg_0889_v2_egn4_Lema_P066270.1	5.22	GC	candidate b-glycosidase
lmctg_0034_v2_egn4_Lema_uP003730.1	4.81	GC	putative SSP-encoding gene
lmctg_1464_v2_egn4_Lema_P106060.1	4.79	GC	putative SSP-encoding gene
lmctg_1143_v2_egn4_Lema_uP081640.1	4.64	GC	putative SSP-encoding gene
lmctg_0029_v2_egn4_Lema_P002510.1	4.62	GC	similar to MFS drug transporter
lmctg_0147_v2_masked_T000001	4.18	GC	putative SSP-encoding gene
lmctg_0147_v2_egn4_Lema_uP015000.1	4.11	GC	similar to aldo-keto reductase (YakC)

lmctg_0978_v2_egn4_Lema_P074930.1	3.95	GC	putative SSP-encoding gene
lmctg_1325_v2_egn4_Lema_P095680.1	3.93	GC	similar to cytochrome P450
lmctg_0901_v2_T000093	3.69	GC	similar to flavin-binding monooxygenase
lmctg_0767_v2_T000002	3.64	GC-island	putative SSP-encoding gene
lmctg_0438_v2_egn4_Lema_P029260.1	3.40	GC	putative SSP-encoding gene
lmctg_0542_v2_egn4_Lema_P034520.1	3.32	GC	similar to integral membrane protein
lmctg_1500_v2_egn4_Lema_P112170.1	3.30	GC	similar to yeast U1 snRNP protein
lmctg_1149_v2_egn4_Lema_P082070.1	3.30	GC	similar to monooxygenase
lmctg_0897_v2_egn4_Lema_uP067030.1	3.23	GC	putative SSP-encoding gene
lmctg_0447_v2_egn4_Lema_P030120.1	3.23	GC	putative SSP-encoding gene
lmctg_0245_v2_egn4_Lema_P019510.1	3.19	GC	similar to putative short-chain dehydrogenase/reductase
lmctg_1489_v2_egn4_Lema_P111320.1	3.15	GC	similar to NADH oxidase
lmctg_1202_v2_egn4_Lema_P084820.1	2.95	GC	putative SSP-encoding gene
lmctg_0377_v2_egn4_Lema_P026200.1	2.92	GC	similar to telomeric DNA binding protein
lmctg_0471_v2_egn4_Lema_P031120.1	2.88	GC	similar to monosaccharide transporter
lmctg_0100_v2_egn4_Lema_uP011970.1	2.82	GC	putative SSP-encoding gene
lmctg_1335_v2_egn4_Lema_uP098370.1	2.82	GC	putative SSP-encoding gene
lmctg_0901_v2_egn4_Lema_uP068540.1	2.77	GC	similar to bodown198
AT12_ext_SuperContig_9_2	2.75	AT-HB	putative SSP-encoding gene
lmctg_1149_v2_egn4_Lema_P082060.1	2.69	GC	similar to AflR
lmctg_0267_v2_egn4_Lema_P020650.1	2.69	GC	candidate galactose oxidase; N-terminal CBM32 module
lmctg_0901_v2_egn4_Lema_P068620.1	2.57	GC	similar to hemolysin-III family protein
lmctg_1249_v2_egn4_Lema_P090030.1	2.52	GC	putative SSP-encoding gene
lmctg_0016_v2_egn4_Lema_P001250.1	2.48	GC	similar to sugar transporter
lmctg_1633_v2_T000001	2.48	AT-B	putative SSP-encoding gene
lmctg_1433_v2_egn4_Lema_P102510.1	2.47	GC	putative SSP-encoding gene
lmctg_1419_v2_egn4_Lema_P101400.1	2.43	GC	similar to monocarboxylate transporter
lmctg_0719_v2_egn4_Lema_P054520.1	2.39	GC	similar to 6-phosphogluconate dehydrogenase family protein
lmctg_0123_v2_T000025	2.37	AT-B	putative SSP-encoding gene
lmctg_1259_v2_egn4_Lema_P091270.1	2.34	GC	candidate cell-wall active enzyme
lmctg_0252_v2_egn4_Lema_P019670.1	2.33	GC	candidate glycosyltransferase
AT10_ext_SuperContig_0_6	2.33	AT-HB	putative SSP-encoding gene

lmctg_1605_v2_egn4_Lema_P120740.1	2.32	GC	candidate b-glycosidase related to endo-b-1,4-glucanases
lmctg_1478_v2_egn4_Lema_P109080.1	2.25	GC	similar to porphobilinogen deaminase
lmctg_1567_v2_egn4_Lema_P117600.1	2.22	GC	similar to short-chain dehydrogenase
lmctg_0758_v2_egn4_Lema_P058140.1	2.20	AT-B	similar to transporter
lmctg_0089_v2_egn4_Lema_P009780.1	2.20	GC	putative SSP-encoding gene
lmctg_1032_v2_egn4_Lema_P076570.1	2.19	GC	similar to sugar transporter
lmctg_1455_v2_egn4_Lema_P104360.1	2.16	GC	similar to protein tyrosine phosphatase
lmctg_0620_v2_egn4_Lema_P046190.1	2.10	GC	candidate endo-1,4-b-glucanase
lmctg_0497_v2_egn4_Lema_P032200.1	2.08	GC	similar to mitochondrial ribosomal protein subunit L31
lmctg_0874_v2_egn4_Lema_P063060.1	1.99	GC	similar to 3-dehydroquinate synthase
lmctg_1508_v2_T000012	1.97	GC	putative SSP-encoding gene
lmctg_1564_v2_egn4_Lema_P117320.1	1.94	GC	similar to phosphate permease
lmctg_1532_v2_egn4_Lema_P115030.1	1.90	GC	similar to cupin 2 domain-containing protein
lmctg_1370_v2_egn4_Lema_uP099860.1	1.90	GC	putative SSP-encoding gene
lmctg_1464_v2_egn4_Lema_P105930.1	1.83	GC	putative SSP-encoding gene
lmctg_0236_v2_egn4_Lema_P018900.1	1.82	GC	putative SSP-encoding gene
lmctg_0578_v2_egn4_Lema_P041430.1	1.82	GC	similar to legume lectin beta domain protein
lmctg_0904_v2_egn4_Lema_P070100.1	1.81	GC	candidate chitin-binding protein
lmctg_0328_v2_egn4_Lema_P023320.1	1.80	GC	similar to MFS transporter
lmctg_0618_v2_egn4_Lema_P045900.1	1.78	GC	putative SSP-encoding gene
lmctg_1067_v2_egn4_Lema_P078170.1	1.74	GC	similar to tRNA isopentenyltransferase
lmctg_1461_v2_egn4_Lema_P105130.1	1.73	GC	similar to zinc-binding oxidoreductase
lmctg_1182_v2_egn4_Lema_P083680.1	1.70	GC	candidate chitin-binding protein; two CBM18 modules
lmctg_0553_v2_egn4_Lema_P036390.1	1.68	GC	candidate cell-wall active enzyme
lmctg_0061_v2_egn4_Lema_P006020.1	1.66	GC	putative SSP-encoding gene
lmctg_0592_v2_egn4_Lema_P043190.1	1.66	GC	similar to potential oxidoreductase
lmctg_0973_v2_egn4_Lema_uP074680.1	1.66	GC	putative SSP-encoding gene

lmctg_0064_v2_egn4_Lema_P006330.1	1.61	GC	similar to copper homeostasis protein cutc
lmctg_1467_v2_egn4_Lema_P106660.1	1.61	GC	similar to zinc alcohol dehydrogenase
lmctg_0347_v2_egn4_Lema_P024250.1	1.59	GC	similar to mechanosensitive ion channel family protein
AT21_ext_SuperContig_1_33	1.59	AT-B	putative SSP-encoding gene
lmctg_0635_v2_egn4_Lema_P047770.1	1.58	GC	similar to WSC domain protein
lmctg_0040_v2_egn4_Lema_P004710.1	1.57	AT-B	candidate GDP-Man a-mannosyltransferase
lmctg_0948_v2_egn4_Lema_P073930.1	1.57	GC	candidate chitin deacetylase; three CBM18 modules
lmctg_1248_v2_egn4_Lema_P089840.1	1.55	GC	candidate esterase related to cutinases

[a] Only genes with a predicted or known function are presented.

[b] Genes with more than 1.5-fold change in transcript level and an associated p value<0.05 were considered as significantly up-regulated the silenced-*LmDIM5* transformant compared to the wild type v23.1.3 isolate in axenic culture.

[c] GC refers to GC-isochores; AT-HB refers to AT-isochores; AT-B refers to 859(±385) bp transition regions between AT-isochores and GC-isochores; GC-islands refer to regions of more than 1 kb within AT-isochores with a GC content > 50%.
