# Supplementary material for: Epigenetic Control of Effector Gene Expression in the Plant Pathogenic Fungus Leptosphaeria maculans
Source: PLoS Genet. 2014 Mar 6;10(3):e1004227. doi: 10.1371/journal.pgen.1004227 (PMC3945186; doi:10.1371/journal.pgen.1004227)
Supplement: Table S4 — List of L. maculans genes up-regulated in a silenced-LmHP1 background. (PDF) [file pgen.1004227.s005.pdf]

Table S4. List of *L. maculans* genes up-regulated in a silenced-*LmHP1* background

SEQ_ID [a]	Fold change [b]	Location[c]	Function
lmctg_1214_v2_egn4_Lema_P086290.1	8.80	AT-HB	AvrLm4-7
lmctg_1031_v2_egn4_Lema_P076380.1	8.72	AT-B	putative SSP-encoding gene
AT10_ext_SuperContig_10_G00134	7.74	AT-HB	putative SSP-encoding gene
lmctg_0001_v2_T000001	6.65	AT-HB	putative SSP-encoding gene
lmctg_1143_v2_egn4_Lema_uP081640.1	6.17	GC	putative SSP-encoding gene
AT15_ext_SuperContig_5_3	5.97	AT-HB	putative SSP-encoding gene
lmctg_1325_v2_egn4_Lema_P095680.1	5.75	GC	similar to cytochrome P450
AvrLm1	5.60	AT-HB	AvrLm1
lmctg_0898_v2_T000001	5.31	AT-HB	putative SSP-encoding gene
lmctg_1187_v2_egn4_Lema_P084060.1	5.31	GC	similar to NRPS-like enzyme
lmctg_0787_v2_egn4_Lema_P059000.1	5.29	GC	similar to beta-glucosidase
lmctg_1149_v2_egn4_Lema_P081940.1	5.02	GC	similar to aspartate aminotransferase
lmctg_1149_v2_egn4_Lema_P081950.1	5.00	GC	similar to nor-1
lmctg_1228_v2_T000001	4.62	AT-B	putative SSP-encoding gene
lmctg_1149_v2_egn4_Lema_P082020.1	4.16	GC	similar to o-methyltransferase
lmctg_1149_v2_egn4_Lema_P082010.1	4.12	GC	similar to protein TOXD
lmctg_1306_v2_egn4_Lema_P093380.1	4.11	GC	similar to meiotic recombination protein dmc1
lmctg_1587_v2_egn4_Lema_uP119060.1	4.05	AT-HB	AvrLm11
lmctg_0758_v2_egn4_Lema_P058140.1	3.77	AT-B	similar to transporter
lmctg_0329_v2_egn4_Lema_uP023430.1	3.75	AT-HB	putative SSP-encoding gene
lmctg_0245_v2_egn4_Lema_P019510.1	3.56	GC	similar toputative short-chain dehydrogenase/reductase
lmctg_1259_v2_egn4_Lema_P091280.1	3.42	GC	similar to ribonuclease H
AT11_ext_SuperContig_12_8	3.42	AT-B	putative SSP-encoding gene

lmctg_0569_v2_egn4_Lema_P040170.1	3.40	AT-B	similar to proline oxidase
lmctg_0901_v2_egn4_Lema_P068810.1	3.35	GC	similar to high-affinity methionine permease
lmctg_0873_v2_T000014	3.26	GC	similar to Zn-dependent hydrolases of the beta-lactamase fold
lmctg_0978_v2_egn4_Lema_P074930.1	3.10	GC	putative SSP-encoding gene
AT01_ext_SuperContig_0_1	3.06	AT-HB	putative SSP-encoding gene
lmctg_0810_v2_egn4_Lema_P060230.1	3.05	GC	similar to monosaccharide transporter
lmctg_0635_v2_egn4_Lema_P047750.1	3.02	GC	similar to pectinesterase
lmctg_1487_v2_egn4_Lema_P110770.1	3.00	AT-B	similar to transporter
lmctg_1229_v2_egn4_Lema_P086540.1	3.00	AT-HB	similar to Six5
lmctg_1149_v2_egn4_Lema_P081930.1	2.98	GC	similar to salicylate hydroxylase
lmctg_0105_v2_egn2_Lema_P012060.1	2.97	AT-HB	putative SSP-encoding gene
lmctg_1246_v2_egn4_Lema_P089620.1	2.95	GC	similar to NADP-dependent leukotriene B4 12-hydroxydehydrogenase
lmctg_0901_v2_egn4_Lema_P068620.1	2.95	GC	similar to hemolysin-III family protein
lmctg_1547_v2_egn4_Lema_P116280.1	2.87	GC	similar to phosphoglycerate mutase
lmctg_0548_v2_egn4_Lema_P035480.1	2.84	GC	similar to DNA mismatch repair protein mlh1
lmctg_0252_v2_egn4_Lema_P019640.1	2.80	GC	similar to carbonic anhydrase
lmctg_1253_v2_egn4_Lema_P090430.1	2.79	GC	similar to sulfate permease
lmctg_0433_v2_egn4_Lema_P028910.1	2.75	GC	similar to short-chain dehydrogenase/reductase
lmctg_0532_v2_egn4_Lema_P033090.1	2.71	AT-B	putative SSP-encoding gene
lmctg_0122_v2_egn4_Lema_P013520.1	2.71	GC	candidate b-fructosidase
lmctg_1477_v2_egn4_Lema_P108350.1	2.60	GC	similar to dimethylglycine dehydrogenase, mitochondrial precursor
lmctg_1149_v2_egn4_Lema_P081920.1	2.59	GC	similar to polyketide synthase
lmctg_0620_v2_egn4_Lema_P046190.1	2.58	GC	candidate endo-1,4-b-glucanase
lmctg_1259_v2_egn4_Lema_P091270.1	2.57	GC	candidate cell-wall active enzyme

lmctg_0876_v2_egn4_Lema_P063470.1	2.57	GC	similar to CoA ligase
lmctg_1616_v2_egn4_Lema_P122130.1	2.50	AT-B	candidate glycosyltransferase related to Dol-P-Man: a-mannosyltransferases
lmctg_0885_v2_egn4_Lema_P065240.1	2.47	GC	similar to short-chain dehydrogenase
lmctg_1524_v2_egn4_Lema_P113920.1	2.47	GC	similar to FAD binding oxidoreductase
lmctg_1253_v2_egn4_Lema_P090600.1	2.45	GC	similar to aldehyde dehydrogenase
lmctg_1149_v2_egn4_Lema_P082000.1	2.45	GC	similar to short chain dehydrogenase
lmctg_0898_v2_egn4_Lema_uP067380.1	2.43	GC	putative SSP-encoding gene
lmctg_0448_v2_egn4_Lema_uP030130.1	2.41	GC	putative SSP-encoding gene
lmctg_1522_v2_egn4_Lema_P113200.1	2.41	GC-island	putative SSP-encoding gene
lmctg_1464_v2_egn4_Lema_P106060.1	2.41	GC	putative SSP-encoding gene
lmctg_0597_v2_egn4_Lema_P043790.1	2.35	GC	similar to glutathione S-transferase
lmctg_0701_v2_egn4_Lema_P051060.1	2.34	GC	candidate b-glycosidase related to xylanases
lmctg_0876_v2_egn4_Lema_P063460.1	2.33	GC	similar to thioesterase family protein
lmctg_1611_v2_egn4_Lema_P121290.1	2.32	GC	similar to RING and UBP finger domain protein
lmctg_0810_v2_egn4_Lema_P060220.1	2.32	GC	similar to sugar transporter
lmctg_0207_v2_egn4_Lema_P017440.1	2.30	GC	similar to ATP-dependent RNA helicase dbp8
lmctg_0610_v2_egn4_Lema_P045240.1	2.28	GC	similar to oxidase
lmctg_0351_v2_egn4_Lema_P024830.1	2.24	GC	similar to origin recognition complex subunit 3
lmctg_1248_v2_egn4_Lema_P089820.1	2.21	GC	similar to membrane protein
lmctg_1489_v2_egn4_Lema_P111290.1	2.20	GC	similar to zinc-binding oxidoreductase ToxD
lmctg_0607_v2_egn4_Lema_P044980.1	2.19	GC	similar to branched-chain-amino-acid aminotransferase
lmctg_0816_v2_egn4_Lema_P060550.1	2.18	GC	similar to MFS monocarboxylate transporter

lmctg_1452_v2_egn4_Lema_P103620.1	2.17	GC	similar to zinc-binding oxidoreductase ToxD
lmctg_0901_v2_egn4_Lema_P068610.1	2.16	GC	similar to indole-diterpene biosynthesis protein PaxU
lmctg_1148_v2_egn4_Lema_P081840.1	2.15	GC	similar to gliotoxin biosynthesis protein GliK
lmctg_0347_v2_egn4_Lema_P024250.1	2.14	GC	similar to mechanosensitive ion channel family protein
lmctg_1545_v2_egn4_Lema_P116080.1	2.13	GC	similar to MFS sugar transporter
lmctg_1346_v2_egn4_Lema_P099100.1	2.12	GC	similar to pectate lyase
lmctg_1451_v2_egn4_Lema_P103310.1	2.12	GC	candidate b-1,4-xylanase
lmctg_0444_v2_egn4_Lema_P030050.1	2.11	GC	similar to nitrilase
lmctg_1523_v2_egn4_Lema_P113300.1	2.10	GC	similar to phosphoadenosine phosphosulfate reductase
lmctg_0034_v2_egn4_Lema_uP003730.1	2.09	GC	putative SSP-encoding gene
lmctg_0440_v2_egn4_Lema_P029410.1	2.07	GC	similar to polysaccharide deacetylase family protein
lmctg_0372_v2_egn4_Lema_P025790.1	2.07	GC	similar to cystathionine-gamma-lyase
lmctg_0113_v2_egn4_Lema_P012870.1	2.06	GC	similar to stress responsive A/B barrel domain protein
lmctg_1089_v2_egn4_Lema_P078840.1	2.06	GC	similar to glutamate-cysteine ligase
lmctg_0567_v2_egn4_Lema_P039550.1	2.06	GC	similar to citrate synthase
lmctg_1245_v2_T000037	2.06	GC	similar to aldehyde reductase
lmctg_0635_v2_egn4_Lema_P047780.1	2.03	GC	similar to dienelactone hydrolase family protein
lmctg_0950_v2_egn4_Lema_P074030.1	2.03	GC	similar to riboflavin synthase
lmctg_0750_v2_egn4_Lema_P057790.1	2.02	GC	similar to gi|215261417|pdb|3F4A|A Chain A
lmctg_1256_v2_egn4_Lema_P090870.1	2.01	GC	similar to acyl-coa dehydrogenase
lmctg_0398_v2_egn4_Lema_P027290.1	2.01	GC	similar to acetyl-CoA acetyltransferase
lmctg_1467_v2_egn4_Lema_P106940.1	2.01	AT-HB	putative SSP-encoding gene
lmctg_1633_v2_T000001	2.00	AT-B	putative SSP-encoding gene
lmctg_1525_v2_egn4_Lema_P114770.1	1.98	AT-B	similar to amidohydrolase

lmctg_0089_v2_egn4_Lema_P009780.1	1.94	GC	putative SSP-encoding gene
lmctg_0510_v2_egn4_Lema_P032600.1	1.93	AT-B	similar to subtilisin-like protease
lmctg_1432_v2_egn4_Lema_P102280.1	1.92	GC	similar to DNA mismatch repair protein msh3
lmctg_1147_v2_egn4_Lema_P081800.1	1.91	GC	similar to cytochrome P450
lmctg_0901_v2_T000097	1.91	GC	similar to oxidoreductase
lmctg_1324_v2_egn4_Lema_P095110.1	1.90	GC	putative SSP-encoding gene
lmctg_0732_v2_egn4_Lema_P056280.1	1.90	GC	similar to elongin C
lmctg_0885_v2_egn4_Lema_P065260.1	1.89	GC	distantly related to cell-wall active enzymes
lmctg_1147_v2_egn4_Lema_uP081770.1	1.89	GC	similar to cytochrome P450 monooxygenase
lmctg_0632_v2_egn4_Lema_P047320.1	1.89	GC	putative SSP-encoding gene
lmctg_1451_v2_egn4_Lema_P103300.1	1.88	GC	putative SSP-encoding gene
lmctg_0567_v2_egn4_Lema_P039540.1	1.88	GC	similar to isocitrate lyase
lmctg_0471_v2_egn4_Lema_P031200.1	1.88	GC	similar to nitroreductase family protein
lmctg_0373_v2_egn4_Lema_P025890.1	1.87	GC	candidate glycosyltransferase related to Dol-P-Man: a-mannosyltransferases
lmctg_0649_v2_egn4_Lema_P048770.1	1.87	GC	similar to serine/threonine protein kinase
lmctg_0728_v2_egn4_Lema_uP055400.1	1.86	GC	putative SSP-encoding gene
lmctg_1523_v2_egn4_Lema_P113240.1	1.86	AT-B	similar to onanonoxo-7-onima-8-eninoihtemlysoneda
lmctg_1296_v2_egn4_Lema_P092560.1	1.86	GC	similar to 5-methyltetrahydropteroyltriglutamate-homocysteine methyltransferase
lmctg_0498_v2_egn4_Lema_P032300.1	1.85	GC	candidate b-1,4-mannanase
lmctg_0869_v2_egn4_Lema_P062660.1	1.85	GC	similar to peptidyl-prolyl-cis-trans isomerase
lmctg_0346_v2_egn4_Lema_P024090.1	1.85	GC	similar to aromatic aminotransferase Aro8
lmctg_1461_v2_egn4_Lema_P105130.1	1.85	GC	similar to zinc-binding oxidoreductase

lmctg_1261_v2_egn4_Lema_P091390.1	1.84	GC	similar to DNA repair protein rad50
lmctg_1477_v2_egn4_Lema_P108520.1	1.84	GC	similar to DNA repair protein rad5
lmctg_1336_v2_egn4_Lema_P098690.1	1.83	GC	similar to HIT domain protein
lmctg_1220_v2_egn4_Lema_P086490.1	1.83	GC	similar to fumarylacetoacetate hydrolase family protein
lmctg_0093_v2_egn4_Lema_P011090.1	1.83	GC	similar to succinyl-CoA:3-ketoacid-coenzyme A transferase
lmctg_0732_v2_egn4_Lema_P056290.1	1.83	AT-B	similar to membrane protein
lmctg_1428_v2_egn4_Lema_P102050.1	1.82	GC	similar to F-box domain-containing protein
lmctg_1148_v2_egn4_Lema_P081850.1	1.81	GC	similar to dipeptidase
lmctg_0882_v2_egn4_Lema_P064920.1	1.80	GC	similar to quinate transporter
lmctg_0556_v2_egn4_Lema_P037110.1	1.80	GC	similar to quinone oxidoreductase
lmctg_1104_v2_egn4_Lema_P079710.1	1.79	GC	similar to ssDNA binding protein Ssb3
lmctg_1326_v2_egn4_Lema_P096950.1	1.79	GC	similar to Taurine catabolism dioxygenase TauD
lmctg_1148_v2_egn4_Lema_P081820.1	1.79	GC	similar to cytochrome P450
lmctg_1524_v2_egn4_Lema_P113510.1	1.79	GC	similar to enoyl-CoA hydratase
lmctg_0052_v2_egn4_Lema_P005360.1	1.78	GC	similar to 2-oxoisovalerate dehydrogenase subunit alpha
lmctg_1532_v2_egn4_Lema_P115030.1	1.78	GC	similar to cupin 2 domain-containing protein
lmctg_0800_v2_egn4_Lema_P059520.1	1.77	GC	similar to cytochrome P450
lmctg_1462_v2_egn4_Lema_P105260.1	1.77	GC	similar to nucleolar complex-associated protein
lmctg_0704_v2_egn4_Lema_P051840.1	1.77	GC	similar to DNA ligase
lmctg_1498_v2_egn4_Lema_P111680.1	1.77	GC	similar to 5`-nucleotidase protein
lmctg_0557_v2_egn4_Lema_P037220.1	1.75	GC	similar to enoyl-CoA hydratase/isomerase
lmctg_1466_v2_egn4_Lema_P106390.1	1.75	GC	similar to electron transfer flavoprotein
lmctg_0745_v2_egn4_Lema_P057030.1	1.74	GC	similar to TPA: ER glycosyl hydrolase (Edem)

lmctg_0693_v2_egn4_Lema_P050560.1	1.74	GC	similar to DNA repair protein Rad51
lmctg_1350_v2_egn4_Lema_P099380.1	1.74	GC	similar to aldose reductase
lmctg_0066_v2_egn4_Lema_P006890.1	1.73	GC	similar to thioredoxin
lmctg_0186_v2_egn4_Lema_P016230.1	1.73	GC	similar to glucose-6-phosphate 1-epimerase
lmctg_1524_v2_egn4_Lema_P114440.1	1.73	GC	similar to anaphase-promoting complex subunit
lmctg_0854_v2_egn4_Lema_P061990.1	1.72	GC	similar to enoyl-CoA hydratase
lmctg_0595_v2_egn4_Lema_P043580.1	1.71	GC	similar to structural maintenance of chromosomes 5
lmctg_0469_v2_egn4_Lema_P031000.1	1.71	GC	similar to NAD-specific glutamate dehydrogenase
lmctg_0718_v2_egn4_Lema_P053940.1	1.70	GC	similar to methylene-fatty-acyl-phospholipid synthase
lmctg_0352_v2_egn4_Lema_P025010.1	1.70	GC	similar to carboxymethylenebutenolidase
lmctg_0217_v2_egn4_Lema_P017870.1	1.69	GC	similar to Fungal specific transcription factor
lmctg_1212_v2_egn4_Lema_P085920.1	1.69	GC	putative SSP-encoding gene
lmctg_0915_v2_egn4_Lema_P072650.1	1.69	GC	similar to glutathione S-transferase
lmctg_1467_v2_egn4_Lema_P106620.1	1.68	GC	similar to amino acid permease
lmctg_0016_v2_egn4_Lema_P000970.1	1.68	GC	similar to NADP-dependent alcohol dehydrogenase
lmctg_1331_v2_egn4_Lema_P097850.1	1.67	GC	similar to ran-binding protein
lmctg_0101_v2_egn4_Lema_P012020.1	1.66	AT-B	similar to ATP-dependent RNA helicase dbp9
lmctg_0878_v2_egn4_Lema_P063830.1	1.66	GC	similar to beta-glucosidase
lmctg_1667_v2_egn4_Lema_P124520.1	1.66	GC	similar to norsolorinic acid reductase
lmctg_1621_v2_egn4_Lema_P122980.1	1.64	GC	similar to helicase
lmctg_0014_v2_egn4_Lema_P000850.1	1.63	GC	similar to D-tyrosyl-tRNA(Tyr) deacylase
lmctg_0441_v2_egn4_Lema_P029550.1	1.63	GC	similar to carboxylesterase
lmctg_0490_v2_egn4_Lema_P031800.1	1.63	GC	similar to glutathione S-transferase
lmctg_1148_v2_egn4_Lema_P081860.1	1.63	GC	similar to aminotransferase
lmctg_0553_v2_egn4_Lema_P036430.1	1.62	GC	similar to protein RTM1

lmctg_0279_v2_egn4_Lema_P021480.1	1.62	GC	similar to DNA-directed RNA polymerase I complex (subunit)
lmctg_0112_v2_egn4_Lema_P012740.1	1.61	GC	putative SSP-encoding gene
lmctg_0163_v2_egn4_Lema_P015520.1	1.61	GC	similar to aldehyde reductase I
lmctg_0757_v2_egn4_Lema_P058110.1	1.60	GC	similar to amino acid permease
lmctg_1453_v2_egn4_Lema_P103800.1	1.60	GC	similar to homoserine-O-acetyltransferase
lmctg_1317_v2_egn4_Lema_P093910.1	1.60	GC	similar to dihydrodipicolinate synthetase family protein
lmctg_0848_v2_egn4_Lema_P061720.1	1.60	GC	similar to thioesterase family protein
lmctg_1106_v2_egn4_Lema_P079850.1	1.59	GC	similar to rhomboid family membrane protein
lmctg_0901_v2_egn4_Lema_P068910.1	1.59	GC	similar to nicotinamidase
lmctg_0016_v2_egn4_Lema_P001150.1	1.59	GC	similar to C-1-tetrahydrofolate synthase
lmctg_0468_v2_egn4_Lema_P030960.1	1.59	GC	similar to S-adenosylmethionine synthetase
lmctg_0941_v2_egn4_Lema_P073690.1	1.59	GC	similar to DNA repair protein Nse1
lmctg_0907_v2_egn4_Lema_P071280.1	1.59	GC	similar to arsenite-translocating ATPase
lmctg_1489_v2_egn4_Lema_P111000.1	1.58	GC	putative SSP-encoding gene
lmctg_1500_v2_egn4_Lema_P111920.1	1.58	GC	similar to feruloyl esterase
lmctg_1556_v2_egn4_Lema_P116820.1	1.58	GC	similar to ATP-dependent DNA helicase
lmctg_0440_v2_egn4_Lema_P029440.1	1.57	GC	similar to xanthine dehydrogenase
lmctg_0618_v2_egn4_Lema_P045960.1	1.57	GC	similar to methionine synthase
lmctg_0542_v2_egn4_Lema_P034350.1	1.57	GC	similar to thiamin pyrophosphokinase
lmctg_1453_v2_egn4_Lema_P103830.1	1.56	GC	similar to pyrroline-5-carboxylate reductase
lmctg_0404_v2_egn4_Lema_P028030.1	1.56	GC	candidate glycosidase related to chitinases
lmctg_0746_v2_egn4_Lema_P057190.1	1.56	GC	similar to oxidoreductase
lmctg_1147_v2_egn4_Lema_P081810.1	1.56	GC	similar to NRPS-like enzyme

lmctg_0244_v2_egn4_Lema_P019460.1	1.55	GC	similar toputative lipase
lmctg_0284_v2_egn4_Lema_P021940.1	1.55	GC	similar to acyl-CoA dehydrogenase
lmctg_0305_v2_egn4_Lema_P022710.1	1.54	GC	similar to cyclopropane-fatty-acyl-phospholipid synthase
lmctg_0545_v2_egn4_Lema_P035250.1	1.54	GC	similar to ubiquitin C-terminal hydrolase L3
lmctg_0899_v2_egn4_Lema_P067630.1	1.54	GC	similar to peptidyl-prolyl cis-trans isomerase/cyclophilin
lmctg_0236_v2_egn4_Lema_P018900.1	1.54	GC	putative SSP-encoding gene
lmctg_0068_v2_egn4_Lema_P007230.1	1.54	GC	similar to ATP-dependent RNA helicase dbp3
lmctg_0180_v2_egn4_Lema_P016160.1	1.54	GC	similar to gamma-glutamyltranspeptidase
lmctg_0876_v2_egn4_Lema_P063220.1	1.53	GC	similar to 2-hydroxychromene-2-carboxylate isomerase
lmctg_0150_v2_egn4_Lema_P015230.1	1.53	GC	similar to cytoplasm protein
lmctg_0545_v2_egn4_Lema_P035210.1	1.53	GC	similar to DNA polymerase delta subunit 4
lmctg_0915_v2_egn4_Lema_P072530.1	1.52	GC	similar to uridine nucleosidase
lmctg_1568_v2_egn4_Lema_P117610.1	1.52	GC	similar to DSBA oxidoreductase
lmctg_0293_v2_egn4_Lema_P022300.1	1.52	GC	similar to pyridoxine biosynthesis protein PDX1
lmctg_0374_v2_egn4_Lema_P025910.1	1.51	GC	similar to aminoglycoside phosphotransferase
lmctg_0904_v2_egn4_Lema_P070220.1	1.51	GC	similar to MFS transporter
lmctg_1580_v2_egn4_Lema_P118540.1	1.51	GC	similar to translin-associated factor X
lmctg_0390_v2_egn4_Lema_P026890.1	1.50	GC	similar to voltage-gated potassium channel subunit beta-1 channel subunit beta-1
lmctg_0232_v2_egn4_Lema_P018660.1	1.50	GC	similar to Ricin B lectin

[a] Only genes with a predicted or known function are presented.

[b] Genes with more than 1.5-fold change in transcript level and an associated p value<0.05 were considered as significantly up-regulated the silenced-*LmHP1* transformant compared to the wild type v23.1.3 isolate in axenic culture.

[c] GC refers to GC-isochores; AT-HB refers to AT-isochores; AT-B refers to 859 (±385) bp transition regions between AT-isochores and GC-isochores; GC-islands refer to regions of more than 1 kb within AT-isochores with a GC content > 50%.
