# Supplementary material for: Epigenetic Control of Effector Gene Expression in the Plant Pathogenic Fungus Leptosphaeria maculans
Source: PLoS Genet. 2014 Mar 6;10(3):e1004227. doi: 10.1371/journal.pgen.1004227 (PMC3945186; doi:10.1371/journal.pgen.1004227)
Supplement: Table S5 — List of genes up-regulated in both a silenced-LmDIM5 and in a silenced-LmHP1 background. (PDF) [file pgen.1004227.s006.pdf]

Table S5. List of genes up-regulated in both a silenced-*LmDIM5* and in a silenced-*LmHP1* background

SEQ_ID	Fold change in silenced-*LmHP1* [a,b]	Fold change in silenced-*LmDIM5* [a,b]	Location [c]	Function
lmctg_0001_v2_T000001	6.65	77.16	AT-HB	putative SSP-encoding gene
lmctg_1539_v2_T000001	13.80	67.97	AT-HB	predicted protein
lmctg_0898_v2_T000001	5.31	62.19	AT-HB	putative SSP-encoding gene
lmctg_1467_v2_T000001	11.85	61.03	AT-B	predicted protein
lmctg_1228_v2_T000001	4.62	55.76	AT-B	putative SSP-encoding gene
lmctg_0105_v2_egn2_Lema_P012060.1	2.97	30.18	AT-HB	putative SSP-encoding gene
AT10_ext_SuperContig_10_G00134	7.74	17.45	AT-HB	putative SSP-encoding gene
AvrLm1	5.60	16.93	AT-HB	AvrLm1
lmctg_1214_v2_egn4_Lema_P086290.1	8.80	16.83	AT-HB	AvrLm4-7
lmctg_1031_v2_egn4_Lema_P076380.1	8.72	14.56	AT-B	putative SSP-encoding gene
AT15_ext_SuperContig_5_3	5.97	11.45	AT-HB	putative SSP-encoding gene
lmctg_0732_v2_egn4_Lema_P056150.1	5.92	9.17	GC	hypothetical protein
lmctg_1311_v2_T000008	2.24	8.98	AT-HB	predicted protein
lmctg_0448_v2_egn4_Lema_uP030130.1	2.41	8.91	GC	putative SSP-encoding gene
lmctg_1212_v2_T000025	3.89	8.30	GC	predicted protein
lmctg_1451_v2_egn4_Lema_P103310.1	2.12	8.14	GC	candidate b-1,4-xylanase
lmctg_1229_v2_egn4_Lema_P086540.1	3.00	8.04	AT-HB	similar to Six5
lmctg_0898_v2_egn4_Lema_uP067380.1	2.43	6.94	GC	putative SSP-encoding gene
lmctg_0122_v2_egn4_Lema_P013520.1	2.71	5.96	GC	candidate b-fructosidase
lmctg_0610_v2_egn4_Lema_P045240.1	2.28	5.93	GC	similar to oxidase

lmctg_0810_v2_egn4_Lema_P060230.1	3.05	5.84	GC	similar to monosaccharide transporter
lmctg_0810_v2_egn4_Lema_P060220.1	2.32	5.48	GC	similar to sugar transporter
lmctg_0034_v2_egn4_Lema_uP003730.1	2.09	4.81	GC	putative SSP-encoding gene
lmctg_1464_v2_egn4_Lema_P106060.1	2.41	4.79	GC	putative SSP-encoding gene
lmctg_0869_v2_egn4_Lema_P062620.1	8.21	4.75	GC	predicted protein
lmctg_0550_v2_T000028	1.85	4.69	AT-HB	predicted protein
lmctg_1143_v2_egn4_Lema_uP081640.1	6.17	4.64	GC	putative SSP-encoding gene
lmctg_1459_v2_egn4_Lema_uP104510.1	8.59	4.52	AT-HB	predicted protein
lmctg_1624_v2_egn4_Lema_P123310.1	3.29	4.49	GC	predicted protein
lmctg_0347_v2_egn4_Lema_P024400.1	3.14	4.05	GC	hypothetical protein
lmctg_0978_v2_egn4_Lema_P074930.1	3.10	3.95	GC	putative SSP-encoding gene
lmctg_1325_v2_egn4_Lema_P095680.1	5.75	3.93	GC	similar to cytochrome P450
lmctg_0572_v2_egn4_Lema_P040750.1	2.31	3.66	GC	predicted protein
lmctg_1517_v2_egn4_Lema_P113080.1	1.96	3.40	AT-B	predicted protein
lmctg_1500_v2_T000029	3.49	3.20	GC	predicted protein
lmctg_0245_v2_egn4_Lema_P019510.1	3.56	3.19	GC	similar to putative short-chain dehydrogenase/reductase
lmctg_1250_v2_egn4_Lema_P090200.1	9.99	3.01	GC	hypothetical protein
lmctg_0600_v2_egn4_Lema_P044140.1	1.78	2.97	GC	hypothetical protein
lmctg_1453_v2_egn4_Lema_P104010.1	1.73	2.80	GC	hypothetical protein
lmctg_1149_v2_egn4_Lema_P082080.1	2.22	2.76	GC	hypothetical protein
lmctg_1420_v2_egn4_Lema_P101410.1	1.90	2.71	GC	predicted protein
lmctg_0573_v2_egn4_Lema_uP040760.1	2.44	2.69	GC	predicted protein
lmctg_0204_v2_egn4_Lema_uP017270.1	1.88	2.68	GC	predicted protein
lmctg_0347_v2_egn4_Lema_P024460.1	3.60	2.67	GC	hypothetical protein
lmctg_0901_v2_egn4_Lema_P068620.1	2.95	2.57	GC	similar to hemolysin-III family protein

lmctg_0869_v2_egn4_Lema_P062610.1	4.59	2.56	GC	predicted protein
lmctg_1613_v2_egn4_Lema_P121760.1	1.51	2.52	GC	predicted protein
lmctg_1212_v2_egn4_Lema_uP086140.1	3.58	2.51	GC	predicted protein
lmctg_0024_v2_egn4_Lema_P002220.1	1.83	2.50	GC	predicted protein
lmctg_1633_v2_T000001	2.00	2.48	AT-B	putative SSP-encoding gene
lmctg_1259_v2_egn4_Lema_P091270.1	2.57	2.34	GC	candidate cell-wall active enzyme
lmctg_1508_v2_egn4_Lema_P112670.1	1.66	2.27	GC	hypothetical protein
lmctg_1626_v2_egn4_Lema_uP123840.1	1.63	2.24	AT-HB	predicted protein
lmctg_0442_v2_egn4_Lema_P029790.1	2.22	2.20	GC	predicted protein
lmctg_0758_v2_egn4_Lema_P058140.1	3.77	2.20	AT-B	similar to transporter
lmctg_0089_v2_egn4_Lema_P009780.1	1.94	2.20	GC	putative SSP-encoding gene
lmctg_0757_v2_egn4_Lema_P058120.1	1.73	2.20	GC	hypothetical protein
lmctg_0562_v2_egn4_Lema_uP038240.1	1.77	2.14	AT-HB	predicted protein
lmctg_0620_v2_egn4_Lema_P046190.1	2.58	2.10	GC	candidate endo-1,4-b-glucanase
lmctg_0587_v2_egn4_Lema_uP042640.1	1.76	2.08	GC	predicted protein
lmctg_1244_v2_egn4_Lema_uP088760.1	1.64	1.97	GC	predicted protein
lmctg_1532_v2_egn4_Lema_P115030.1	1.78	1.90	GC	similar to cupin 2 domain-containing protein
lmctg_0236_v2_egn4_Lema_P018900.1	1.54	1.82	GC	putative SSP-encoding gene
lmctg_0560_v2_egn4_Lema_uP037580.1	2.62	1.80	GC	predicted protein
lmctg_0197_v2_egn4_Lema_P017020.1	2.18	1.73	GC	hypothetical protein
lmctg_1691_v2_T000001	1.67	1.73	AT-B	predicted protein
lmctg_1461_v2_egn4_Lema_P105130.1	1.85	1.73	GC	similar to zinc-binding oxidoreductase
lmctg_1042_v2_egn4_Lema_P077130.1	1.69	1.73	GC	hypothetical protein
lmctg_0898_v2_egn4_Lema_P067360.1	1.69	1.66	GC	hypothetical protein

lmctg_0347_v2_egn4_Lema_P024250.1	2.14	1.59	GC	similar to mechanosensitive ion channel family protein

[a] The silenced-*LmHP1* transformant showed 35% residual expression of *LmHP1*; the silenced-*LmDIM5* transformant showed 16% residual expression of *LmDIM5* compared to wild type.

[b] Genes with more than 1.5-fold change in transcript level in axenic culture and an associated p value of <0.05 were considered as significantly up-regulated in the silenced-*LmDIM5* or in the silenced-*LmHP1* transformants compared to wild type.

[c] GC refers to GC-isochores; AT-HB refers to AT-isochores; AT-B refers to the 859 (±385) bp transition region between AT-isochores and GC-isochores.
