# Supplementary material for: Epigenetic Control of Effector Gene Expression in the Plant Pathogenic Fungus Leptosphaeria maculans
Source: PLoS Genet. 2014 Mar 6;10(3):e1004227. doi: 10.1371/journal.pgen.1004227 (PMC3945186; doi:10.1371/journal.pgen.1004227)
Supplement: Table S6 — Number of sequences generated and exploitable following TAIL-PCR experiments on transformants v29.3.1-AvrLm1, v29.3.1-AvrLm6, v29.3.1-LmCys2 and NzT4-AvrLm4-7. (PDF) [file pgen.1004227.s007.pdf]

Table S6. Number of sequences generated and exploitable following TAIL-PCR experiments on transformants v29.3.1-*AvrLm1*, v29.3.1-*AvrLm6*, v29.3.1-*LmCys2* and NzT4-*AvrLm4-7*

Transformants	Number of transfomants tested	Number of sequences generated	Number of exploitable sequences [a]
v29.3.1-*AvrLm1*	11	4	3
v29.3.1-*AvrLm6*	13	10	3
v29.3.1-*LmCys2*	15	3	2
NzT4-*AvrLm4-7*	27	19	8
Total	66	36	16

[a] Number of sequences which had a unique BLAST hit in the genome of *L. maculans* and that were not redundant.
