# Supplementary material for: Epigenetic Control of Effector Gene Expression in the Plant Pathogenic Fungus Leptosphaeria maculans
Source: PLoS Genet. 2014 Mar 6;10(3):e1004227. doi: 10.1371/journal.pgen.1004227 (PMC3945186; doi:10.1371/journal.pgen.1004227)
Supplement: Table S7 — Characteristics of the sequences obtained by TAIL-PCR. (PDF) [file pgen.1004227.s008.pdf]

Table S7. Characteristics of the sequences obtained by TAIL-PCR

Transformants	SC[a]	Isochore type	Location of the insertion
v29.3.1-*AvrLm1*-4	4	GC	Gene encoding for a protein overexpressed during meiosis
v29.3.1-*AvrLm1*-8	0	GC	Gene encoding for a glycosyl hydrolase
v29.3.1-*AvrLm1*-12	8	GC	Gene encoding for a nuclear GTP binding protein
v29.3.1-*AvrLm6*-4	21	GC	Gene with no homology
v29.3.1-*AvrLm6*-5	0	GC	Gene encoding for a protein kinase
v29.3.1-*AvrLm6*-11	2	GC	Gene encoding for a AMP deaminase
v29.3.1-*LmCys2*-4	21	GC	Gene encoding for a hypothetical protein
v29.3.1-*LmCys2*-15	23	GC	Gene encoding for an allantoate permease
NzT4-*AvrLm4-7*-3	4	GC	Intergenic region
NzT4-*AvrLm4-7*-7	18	GC	Intergenic region
NzT4-*AvrLm4-7*-16	7	GC	Intergenic region
NzT4-*AvrLm4-7*-18	3	GC	Gene encoding for a glycosyl hydrolase
NzT4-*AvrLm4-7*-25	10	GC	Intergenic region
NzT4-*AvrLm4-7*-27	1	GC	Gene encoding for a hypothetical protein
NzT4-*AvrLm4-7*-28	6	GC	Promoter of a gene encoding for an aquaporine
NzT4-*AvrLm4-7*-9	10	GC	Promoter of a gene with no homology

[a]SC: Super Contig.
