# Supplementary material for: Epigenetic Control of Effector Gene Expression in the Plant Pathogenic Fungus Leptosphaeria maculans
Source: PLoS Genet. 2014 Mar 6;10(3):e1004227. doi: 10.1371/journal.pgen.1004227 (PMC3945186; doi:10.1371/journal.pgen.1004227)
Supplement: Table S8 — List of PCR primers used in this study. (PDF) [file pgen.1004227.s009.pdf]

Table S8. List of PCR primers used in this study

Primers (5′-3′)	
**TAIL-PCR**	
pPZPnat1-LB1	GTAAAGCCTGGGGTGCCTAATGAG
pPZPnat1-LB2	CAGTCGGGAAACCTGTCGT
pPZPnat1-LB3	GGCGGTTTGCGTATTGG
pBHt2-LB1	GGGTTCCTATAGGGTTTCGCTCATG
pBHt2-LB2	CATGTGTTTGAGCATATAAGAAACCCT
pBHt2-LB3	GAATTAATTCGGCGTTAATTCAGT
pBBH-LB1	CGGCTATTGGTAATAGGACACTGG
pBBH-LB2	CAACCCTCAACTGGAAACGGGCCGGA
pBBH-LB3	TCCAGGGCGTGTGCCAGGTGC
AD2	AGWGNAGWANCAWAGC
**RACE-PCR**	
HP1-5UTRL1	GACTCCATTGCTTCTCCTGCTTTGG
HP1-5UTRL2	CCCTTCCGCTTTTGTCCGCCCTTGG
HP1-3UTRU1	CCAAAGCAGGAGAAGCAATGGAGTC
HP1-3UTRU2	GAGCCACCTCGTCTTCACGCACCC
DIM5-5UTRL1	GCTGCGACCCATCTGCGGAGAG
DIM5-5UTRL2	CGTAGCCTGAGATGTAGCGGAAGC
DIM5-3UTRU1	CGAGGTGAGATTATTACCGACGCC
DIM5-3UTRU2	GTGTCCTACAACAAGCACGACCCG
**q-RT-PCR**	
AvrLm1	GGCCAAAACAGCAGATAAT
	AGGCCATAGCGTAGAAGGTA
AvrLm6	AAACGGCACTATTACGAAAA
	GATTAGGCGAGAAGCAAGT
AvrLm4-7	GCCCTGCATAACTACCGAC
	TCCTGGCCAAATATAACTCC
LmCys2	TCTACTGCCAGGACACAGAC
	ATGAAGCGGAGAATGAGAAT
β-tubulin-RT	AAGAACTCATCCTACTTCGA
	TGAATAGCTCCTGAATGG
actin-RT	AGTGCGATGTCGATGTCAG
	AAGAGCGGTGATTTCCTTCT
LmHP1	TTCGCAGACACCAAGTATG
	AGTAGGCTCGCTTAGAATCG
LmDIM5	CGGACACATACAATGGTG
	TGATGGTAATACGGGGAGTA
β-tubulin	AAGAACTCATCCTACTTCGA
	TGAATAGCTCCTGAATGG

actin	AGTGCGATGTCGATGTCAG
	AAGAGCGGTGATTTCCTTCT
**Construction for pPZPnat1-*LmHP1* and pPZPnat1-*LmDIM5* vectors**	
Silent*LmHP1-Hind*III+	AAGCTTGCCACCCATTTCCCCGAGGACAGC
Silent*LmHP1-Bam*HI-	CCACGTCACTAGACCGACCAAGG
Silent*LmHP1-Bam*HI+	GGATCCGACTCCATTGCTTCTCCTGCTTTGG
Silent*LmHP1-Eco*RI-	GAATTCGCCACCCATTTCCCCGAGGACAGC
Silent*LmDIM5-Hind*III+	AAGCTTCCATCAACTCAGTTCCCACCCCG
Silent*LmDIM5-Bam*HI-	GGATCCGTCCACTGCTCAGGTCCAAAGCC
Silent*LmDIM5-Bam*HI+	GGATCCGGCGTCGGTAATAATCTCACCTCG
Silent*LmDIM5-Xma*I-	CCCGGGCCATCAACTCAGTTCCCACCCCG
**Construction for pBHt2-*LmHP1*-GFP vector**	
*LmHP1-Eco*RI	GAATTCCCTGGGCTTATTCGGTACTG
*LmHP1-Xba*I	TCTAGAGCGAGATGCATGCTCAACGAC
eGFP-*Cla*I	ATCGATATGGTGAGCAAGGGCGAGGAG
C-eGFP-*Spe*I	ACTAGTTTACTTGTACAGCTCGTCCATGC
C- *LmHP1-Cla*I	ATCGATGTAAGCATCGTCCATGGTGAGG
C- *LmHP1-Spe*I	ACTAGTGCCATCGCAATTGAGCC
**ChIP-qPCR**	
AvrLm4-7	GCCCTGCATAACTACCGAC
	TCCTGGCCAAATATAACTCC
AvrLm1	GGCCAAAACAGCAGATAAT
	AGGCCATAGCGTAGAAGGTA
*AvrLm4-7*_PROM	TCGCTAAGCTTGCAAAACG
	AGCTTCCATGTAGAGTAGCTTTCC
*AvrLm1*_PROM	TAATCCATTCCTCACCTCGTGG
	TAGTACTTACGAAGGATGGGAG
H2A	ATGACTGGAGGCAAGTCCGGAG
	AGACTTGTTTTCGCGTGCGCC
